# Optical Coherence Tomography in Schizophrenia Spectrum Disorders: A Systematic Review and Meta-analysis

**DOI:** 10.1016/j.bpsgos.2023.08.013

**Published:** 2023-08-30

**Authors:** William Shew, Daniel J. Zhang, David B. Menkes, Helen V. Danesh-Meyer

**Affiliations:** a Department of Ophthalmology, New Zealand National Eye Centre, University of Auckland, Auckland, New Zealand; b Dunedin School of Medicine, University of Otago, Dunedin, New Zealand; c Department of Psychological Medicine, University of Auckland, Auckland, New Zealand

**Keywords:** Meta-analysis, Optical coherence tomography, Schizophrenia, Schizophrenia spectrum disorder, Systematic review

## Abstract

**Background:**

Inner retinal atrophy has been demonstrated in schizophrenia spectrum disorder (SSD) using optical coherence tomography (OCT). This systematic review and meta-analysis investigated the role of contemporary Fourier domain OCT devices in SSD.

**Methods:**

MEDLINE, PubMed, Scopus, Embase, PsycInfo, PYSNDEX, World Health Organization, and Cochrane databases were searched from inception until May 2022. All peer-reviewed adult SSD case-control studies using Fourier domain OCT were included. Ocular pathologies known to affect retinal OCT scans were excluded. Search, data appraisal, and summary data extraction were independently performed by 2 authors.

**Results:**

The review criteria was met by k = 36 studies, with k = 24 studies (1074 cases, 854 controls) suitable for meta-analysis. The SSD group exhibited a thinner global peripapillary retinal nerve fiber layer (−3.26 μm, 95% CI, −5.07 to −1.45, *I*^2^ = 64%, k = 21), thinner average macular layer (−7.88 μm, 95% CI, −12.73 to −3.04, *I*^2^ = 65%, k = 11), and thinner macular ganglion cell-inner plexiform sublayer (−2.44 μm, 95% CI, −4.13 to −0.76, *I*^2^ = 30%, k = 8) compared with the control group. Retinal nerve fiber layer findings remained significant after exclusion of metabolic disease, low quality, outlier, and influential studies. Studies involving eye examinations to exclude eye disease were associated with greater atrophy in SSD. Except for cardiometabolic disease, most studies did not report clinically significant covariate data known to influence retinal thickness.

**Conclusions:**

Individuals with SSD generally exhibited retinal atrophy, possibly paralleling reduced brain volumes documented in clinical imaging. Prospective longitudinal studies that collect clinical data, including various illness phases, and control for confounders will be necessary to evaluate retinal atrophy as a biomarker in SSD.

Schizophrenia spectrum disorders (SSDs) include schizophrenia, schizoaffective, and schizotypal disorders. Schizophrenia, the most severe form of SSD, affects over 20 million people worldwide (1,2). SSDs are typically diagnosed through comprehensive psychiatric assessments, but neurobiological insights are crucial for objective diagnostic criteria and monitoring of treatment modalities. Identification of biomarkers in SSD is a rapidly evolving field (3); however, there is a lack of consensus about the reliability of these biomarkers and surrogate outcomes in predicting or monitoring disease progression (4).

The retina shares embryological, anatomical, and physiological characteristics with the brain (3,5). The inner retina, including the inner plexiform, ganglion cell, and retinal nerve fiber layers, form a cellular continuum between the eye and the brain through the optic nerve (5). The accessibility of the retina therefore provides a window to neurodegenerative, autoimmune, and inflammatory conditions that affect the brain (5, 6, 7, 8, 9).

Optical coherence tomography (OCT) provides rapid, noninvasive, high-resolution images of the retinal structures and has thus become an appealing candidate for studying neurodegenerative and neuropathological changes. OCT has transitioned from time domain (TD-OCT) to Fourier domain-based techniques that enable faster image acquisition and improved spatial resolutions of up to 2.1 μm (10). The 2 major Fourier domain-based devices are spectral domain OCT (SD-OCT) and swept source OCT devices. Utilizing custom imaging algorithms, swept source OCT allows in vivo noninvasive imaging of the retinal and choroidal vasculature. OCT has demonstrated loss of retinal ganglion cells and their axons in Alzheimer’s disease (11), Parkinson’s disease (12), multiple sclerosis (13, 14, 15), and normal aging. Furthermore, atrophy of the inner retinal layers is significantly correlated with progressive cognitive impairment and brain volume loss (16, 17, 18).

Previous meta-analyses of OCT have demonstrated thinning of the innermost retina layer around the optic nerve head, known as the peripapillary retinal nerve fiber layer (pRNFL), in individuals with schizophrenia compared with controls (19, 20, 21, 22, 23). However, these analyses pooled both TD-OCT and SD-OCT data, which are not interchangeable (24), and have not adequately controlled for confounding factors, including metabolic syndrome, smoking, and disease duration (25). SD-OCT also reliably identifies the ganglion cell-inner plexiform layer (GCIPL) containing ganglion cell bodies, bipolar cell axons, and amacrine cell dendrites, which is sensitive to retrograde synaptic degeneration and other neurodegenerative conditions. The imprecision of TD-OCT may also account for the finding that pRNFL thinning has been shown in SSD, while the macula, which incorporates a smaller percentage of the retinal nerve fiber layer, has not. This meta-analysis aims to address the limitations of previous analyses and to update available evidence of Fourier domain OCT imaging in individuals with SSDs compared with control participants.

## Methods and Materials

This systematic review was registered in PROSPERO (CRD42022297757) and reported under Preferred Reporting Items for Systematic Reviews and Meta-analyses (PRISMA) guidelines (26). Initial searches of review protocols found a single protocol (PROSPERO CRD42018109344) related to an existing published meta-analysis (19).

### Inclusion Criteria

Peer-reviewed published studies using Fourier domain OCT comparing individuals with SSDs with control participants aged 16 to 65 years were included. SSDs included schizophrenia, schizoaffective disorder, and schizotypal personality disorder as classified by the DSM-5 or ICD-10 and related legacy versions.

### Exclusion Criteria

Schizophreniform and brief psychotic disorders were excluded as they may not progress to chronic forms of SSD. Studies with TD-OCT, pathologies known to affect retinal OCT scans (e.g., glaucoma, diabetic macular edema), and high refractive error (>6 dioptres) were excluded. Case reports and case series with <10 cases were excluded due to risk of bias.

### Search Methods

MEDLINE, PubMed, Scopus, Embase, PsycINFO, PYSNDEX, CENTRAL, and the World Health Organization International Clinical Trials Registry Platform were searched using the Boolean phrase (schizophrenia OR schizoaffective OR psychosis OR psychiatry OR psychiatric) AND (“optical coherence tomography” OR “ganglion cell” OR retina∗). Medical subject heading terms were used if available. Studies from inception to May 2022 were included with no restrictions on setting, country, or language. Reference lists were also manually searched. Gray literature was also searched for articles that fit the eligibility criteria. References were managed by EndNote version 20 (Clarivate). Two reviewers (WS, DJZ) independently screened all electronic abstracts and retrieved full-text articles if studies were considered to match the eligibility criteria. Differences in selection were resolved through discussion, and a third reviewer (HVD-M) was available to resolve disagreements.

### Data Extraction and Study Quality Assessment

Summary OCT-derived pRNFL, ETDRS (Early Treatment of Diabetic Retinopathy Study)–standardized subfield macular thickness, macular inner retinal layer submeasures, and disease/patient covariates were extracted (see the Supplement). Values were extracted from the right eye whenever possible to maintain independent sampling. Representative examples of the macular ETDRS grid and pRNFL OCT scans are shown in the supplemental figures. Authors were not contacted for further data. The Newcastle Ottawa Scale (NOS) case-control tool was used for evaluation of study quality (27,28). Data extraction and study quality assessment were independently performed by 2 reviewers (WS, DJZ) and managed on Microsoft Excel version 2112 Build (Microsoft Corporation). Automated data integrity checks were performed between reviewers, and any differences in data extraction or quality assessment were resolved through discussion.

### Statistical Analysis

Statistical analysis was performed using the *metafor* version 3.0.2 (29), *meta* version 5.2.0 (30), and *dmetar* version 0.0.9000 (31) packages (R Foundation). Pooled analyses of mean differences were conducted using inverse variance-weighted random-effects modeling with Knapp and Hartung adjustments (32). *I*^2^ statistics were computed to summarize the proportion of variation across studies that was attributable to heterogeneity rather than random chance, and an *I*^2^ value >50% was considered to indicate moderate to high heterogeneity (33). The tau^2^ estimate of heterogeneity was also calculated using the DerSimonian-Laird estimator (34). Exploration of statistical heterogeneity was performed using standard outlier identification, Baujat plots (35) (using the leave one out method), and graphic display of study heterogeneity plot analysis. Quantification of influential studies was identified with the *InfluenceAnalysis* function and unsupervised machine learning algorithms (K-means, density reachability and connectivity clustering [DBSCAN], and Gaussian mixture models) were applied to the graphic display of study heterogeneity plot analysis. If outliers and influential studies were considered significant, an additional sensitivity analysis was conducted to exclude significant contributors. Meta-regression and subgroup analysis were performed using a mixed-effects model with an a priori analysis using predefined subgroups and a post hoc analysis using multiple meta-regression analyses. Risk of publication bias was assessed by visually inspecting contour-enhanced funnel plots. Egger’s regression test was performed if study k ≥ 10 and corrected using the Duval & Tweedie trim-and-fill method where possible. All tests were 2-tailed, and *p* < .05 was considered significant.

All measurements were assessed for the relative contributions of study year, device, geographic location, and study quality on heterogeneity. Moderator effects were assessed for Positive and Negative Syndrome Scale (PANSS) score, disease duration, chlorpromazine equivalent antipsychotic daily dose (36), body mass index (BMI), smoking status, and cardiometabolic status.

## Results

### Study and Participant Characteristics

**Figure 1:**
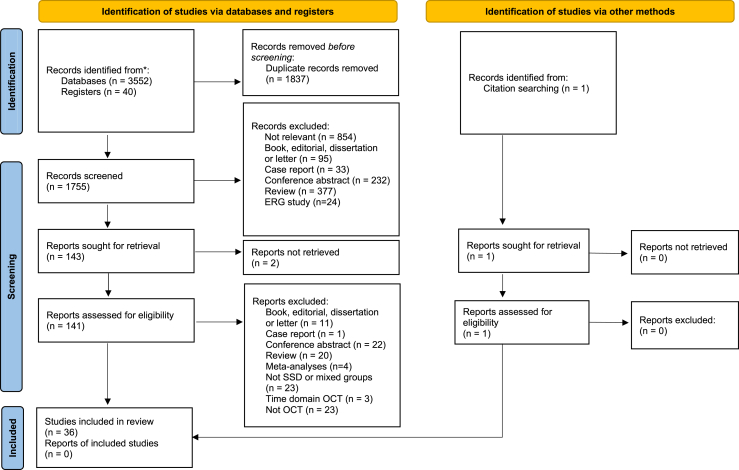
Selection flowchart. No differences were found in study selection after consensus discussion between 2 authors. ERG, electroretinogram; OCT, optical coherence tomography; SSD, schizophrenia spectrum disorder.

A total of 3592 records were identified through database searching. Thirty-six studies were included in qualitative synthesis, and 24 studies were included for meta-analysis (Figure 1). Six studies were considered low quality (NOS ≤ 4) while the rest were of moderate (NOS = 5-6) or high (NOS ≥ 7) quality. In general, it was uncommon (k = 8) for studies to recruit from combined outpatient and inpatient settings, and it was rare (k = 3) to have reported dropout/ineligibility rates. Seven studies did not report or assess whether control participants were free from a previous history of psychiatric disease, and 12 studies recruited control participants from university or clinical staff. Detailed NOS item ratings are included in the Supplement.

A total pool of 1774 cases and 1506 controls matched the selection criteria; of these, 1074 cases and 854 controls were suitable for meta-analysis. In the meta-analysis, the mean age was 38.4 ± 11.7 years for cases and 37.3 ± 11.2 for controls, and the proportion of male participants was 64.8% for cases and 60.2% for controls. For SSD cases, the mean reported duration of illness was 16.1 ± 12.6 years (*n* = 840); the mean PANSS score was 72.3 ± 20.9 (*n* = 381); and the chlorpromazine equivalent dose was 270 ± 368 mg/day (*n* = 474).

Covariate data were incomplete in most studies. Pooled analysis of available data found that the SSD group had no significant statistical difference in the relative risk (RR) of smoking (RR 1.63, 95% CI, 0.84 to 3.19, *I*^2^ = 83%, k = 5, SSD = 123/397, control = 63/302) and cardiometabolic disease (RR 1.04, 95% CI, 0.61 to 1.77, *I*^2^ = 0%, k = 29, SSD = 20/936, control = 17/699). Cardiometabolic disease was usually excluded in studies. A significantly higher mean BMI difference was observed in the SSD group by +1.43 (95% CI, 0.22 to 2.65, *I*^2^ = 59%, k = 6, *n*_SSD_ = 400, *n*_control_ = 310). Tables of study and participant characteristics are included in the Supplement.

### Peripapillary Retinal Nerve Fiber Layer

**Figure 2:**
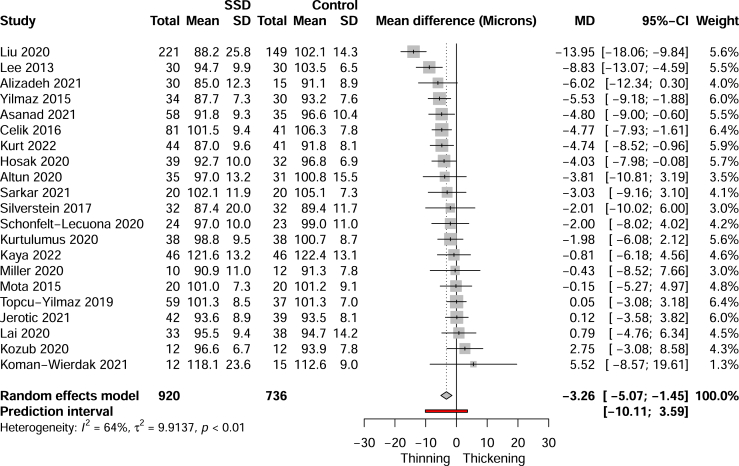
Forest plot of pooled global peripapillary retinal nerve fiber layer thickness. MD, mean difference; SSD, schizophrenia spectrum disorder.

Pooled analysis found significant global pRNFL atrophy of −3.26 μm (95% CI, −5.07 to −1.45, *I*^2^ = 64%, k = 21) in SSD cases compared with controls (Figure 2). Heterogeneity analysis (see the Supplement) had found that Liu *et al.* (37) was a significant outlier and a highly influential study. Exclusion of this study continued to show statistically significant thinning of −2.76 μm (95% CI, −4.15 to −1.36, *I*^2^ = 34%, k = 20), and heterogeneity was no longer statistically significant (*p* = .07).

The sensitivity and subgroup analyses are shown in Table 1. Restriction to high-quality studies (NOS score ≥7) continued to show a significant thinning of pRNFL in SSD cases (*p* = .0006) even after the removal of outliers and highly influential studies (*p* = .027). Significant thinning was also seen in studies with pure schizophrenia cases (*p* = .006) or no cardiometabolic disease (*p* = .005). No significant thinning was observed with mean disease duration < 5 years (*p* = .79) or with mean BMI < 30 (*p* = .17). Insufficient studies were available for pooling when restricted to matched smoking status and untreated cases. Significant differences were found between studies grouped by country (*p* = .021). Asian studies exhibited the greatest degree of mean thinning of −8.89 μm (95% CI, −16.28 to −1.50, *I*^2^ = 70%, k = 4). After removal of the outlier/influential Asian study (37), the analysis continued to demonstrate almost 3 times the pooled average thinning of other continents (−6.48 μm [95% CI, −13.86 to 0.90, *I*^2^ = 16%, k = 3]); however, the difference failed to reach statistical significance. Studies with eye exams found significant SSD pRNFL atrophy while those without eye exams did not.

**Table 1:** Sensitivity and Subgroup Analysis for Global Peripapillary Retinal Nerve Fiber Layer Thinning Comparing Schizophrenia Spectrum Disorder Cases Versus Controls

Subgroup	Studies	Effect size, μm [95% CI]	*p*	*I*^*2*^
Main Analysis	21	−3.26 [−5.07 to −1.45]	.001^a^	64%
Outlier and Influential Cases Removed	20	−2.76 [−4.15 to −1.36]	.0006^a^	34%
High-Quality Studies (NOS Score ≥ 7)	8	−5.00 [−9.06 to −0.93]	.023^a^	77%
High-Quality Studies (NOS Score ≥ 7) and Outliers/Influential Cases Removed	7	−3.68 [−6.77 to −0.60]	.027^a^	44%
Schizophrenia Cases Only	13	−4.00 [−6.62 to −1.38]	.006^a^	71%
Mean Disease Duration < 5 Years	2	−1.20 [−45.72 to 43.31]	.79	16%
No Antipsychotic Treatment	–	Insufficient studies	–	–
No Cardiometabolic Disease	15	−3.66 [−6.03 to −1.29]	.005^a^	71%
Mean Body Mass Index < 30	6	−3.90 [−10.13 to 2.33]	.17	81%
Matched Smoking Status	–	Insufficient studies	–	–
Country				
Asia	4	−8.89 [−16.28 to −1.50]	.021^a^	70%
Europe	13	−2.42 [−3.88 to −0.97]		14%
America	3	−2.42 [−9.99 to 5.14]		20%
Russia	1	2.75 [−4.03 to 9.53]		–
Setting				
Inpatient	5	−4.97 [−13.35 to 3.40]	.64^a^	87%
Outpatient	13	−2.43 [−3.77 to −1.11]		0%
Both	3	−4.10 [−18.03 to 9.83]		80%
Device				
Cirrus	10	−3.80 [−6.00 to −1.60]	.74^a^	40%
RTVue	2	−1.30 [−53.88 to 51.28]		26%
Spectralis	5	−1.84 [−4.38 to 0.70]		22%
Optopol	1	−0.81 [−9.00 to 7.38]		–
3D OCT	2	−6.72 [−111.85 to 98.41]		95%
Spectral OCT	1	−3.03 [−11.73 to 5.67]		–
Eye Exam				
Yes	14	−3.70 [−6.37 to −1.03]	.35^a^	74%
No	7	−2.32 [−12.94 to 0.38]		0%

3D, 3-dimensional; NOS, Newcastle Ottawa Scale; OCT, optical coherence tomography.

a Statistically significant.

Meta-regression with mixed-effects modeling found that the average SSD group age was predictive of increasing global pRNFL atrophy compared with that of the control group by −0.36 μm/year of age (95% CI, −0.64 to −0.07, *I*^2^ = 53%, k = 21). Other variables were not predictive of global pRNFL thinning in SSDs with or without outliers/influential cases. The variables examined included proportion of male patients (*p* = .91), PANSS score (*p* = .33), disease duration (*p* = .57), chlorpromazine equivalent dose (*p* = .31), patient BMI (*p* = .46), publication year (*p* = .95), or NOS score (*p* = .11). Limitations of this model include assuming a linear relationship and a similar spread of pRNFL thinning across the range of variables. There was an insufficient number of studies to fit a meta-regression model for smoking and cardiometabolic status. Post hoc multivariate modeling ranked the most important predictors of global pRNFL thinning in descending order: geographic continent, average patient age, publication year, NOS score, proportion of male patients, clinical setting, and OCT device. PANSS score, disease duration, chlorpromazine equivalent dose, smoking, BMI, and cardiometabolic status could not be included in this model due to incomplete data.

**Figure 3:**
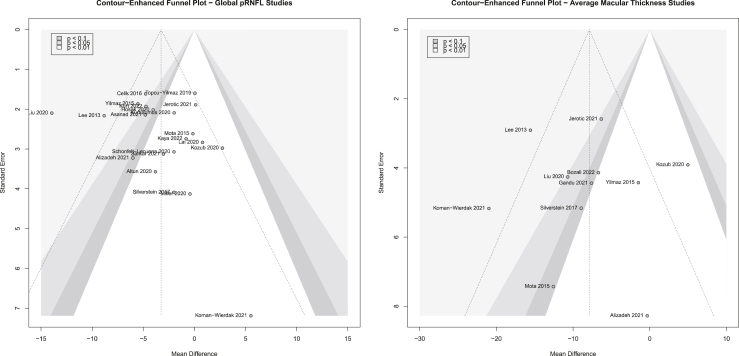
Contour-enhanced funnel plot for global peripapillary retinal nerve fiber layer (pRNFL) thickness and average macular thickness studies.

The contour-enhanced funnel plot (Figure 3) suggests that the previously identified outlier/influential study (37) may reflect publication bias. However, statistical analysis did not detect any plot asymmetry (*p* = .33).

**Figure 4:**
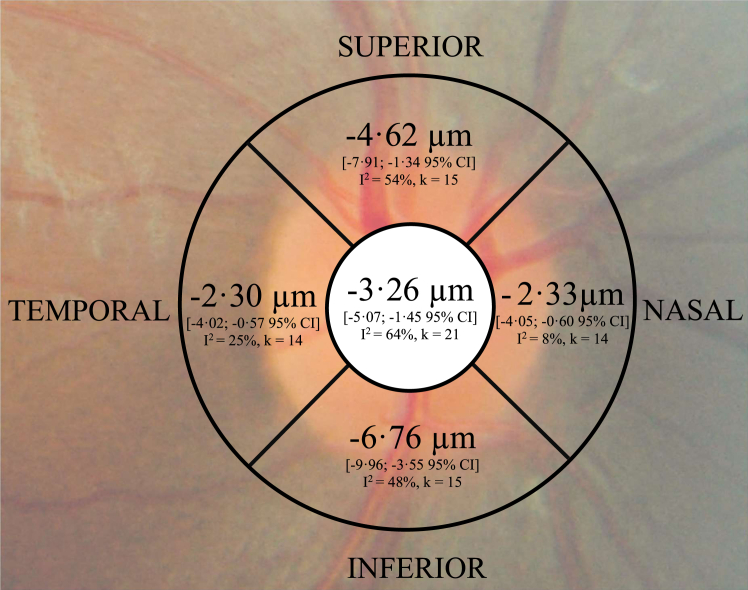
Meta-analysis of right eye of sector peripapillary retinal nerve fiber layer difference between schizophrenia spectrum disorder cases and controls. Central value represents the global measure.

All quadrants had significant thinning of SSD cases compared with controls, with the most severe and consistent thinning seen in the inferior quadrant (Figure 4). Exclusion of outliers/influential studies did not result in loss of statistical significance in any quadrant. Mixed-effect modeling showed sporadic significant moderator effects: disease duration for superior (*p* = .023) and inferior (*p* = .031) quadrants and patient mean age for inferior quadrants (*p* = .0086). After exclusion of outliers/influential studies (37,38), these moderator effects did not remain statistically significant.

Significant publication bias was identified for superior (*p* = .0096) and inferior (*p* = .013) quadrants, which persisted even after elimination of outliers/influential studies (37,38). Correction of publication bias with imputed studies yielded almost 50% greater estimates of SSD thinning in these quadrants (−6.71 μm [95% CI, −10.19 to −3.24, *I*^2^ = 59%, k = 15 + 5 imputed] for the superior and −9.78 μm [95% CI, −13.33 to −6.24, *I*^2^ = 62%, k = 15 + 6 imputed] for the inferior quadrants).

### Macular Thickness

**Figure 5:**
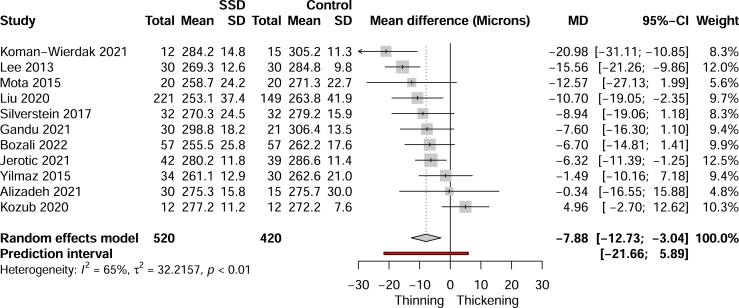
Forest plot of pooled average macular thickness. MD, mean difference; SSD, schizophrenia spectrum disorder.

The SSD group had significantly thinner average macular thickness values, with a mean difference of −7.88 μm (95% CI, −12.73 to −3.04, *I*^2^ = 65%, k = 11) compared with the control group (Figure 5). Heterogeneity analysis (see the Supplement) had found that Koman-Wierdak *et al.* (39), Kozub *et al.* (40), and Lee *et al.* (38) were significant outliers. These studies contained SSD cohorts with mostly acute episodes while the rest consisted of mostly stable SSD cases. Exclusion of these studies continued to show statistically significant thinning in SSD of −6.79 μm (95%, CI −9.41 to −4.18, *I*^2^ = 0%, k = 8) with no residual heterogeneity.

Meta-regression with mixed-effects modeling found that higher quality studies were significantly correlated (coefficient −3.08, 95% CI, −5.03 to −1.14, *p* = .006, k = 11) with macular thinning (see the Supplement). Other variables examined included continent (*p* = .21), OCT device (*p* = .43), setting (*p* = .61), publication year (*p* = .51), mean patient age (*p* = .96), proportion of male cases (*p* = .35), patient BMI (*p* = .45), disease duration (*p* = .78), and chlorpromazine equivalent dose (*p* = .74). There was an insufficient number of studies to fit a meta-regression model for PANSS score, smoking, and cardiometabolic status.

The contour-enhanced funnel plot (Figure 3) suggests that the 3 statistical outlier/influential cases (38, 39, 40) possibly reflect publication bias; however, statistical analysis did not detect plot asymmetry (*p* = .89).

**Figure 6:**
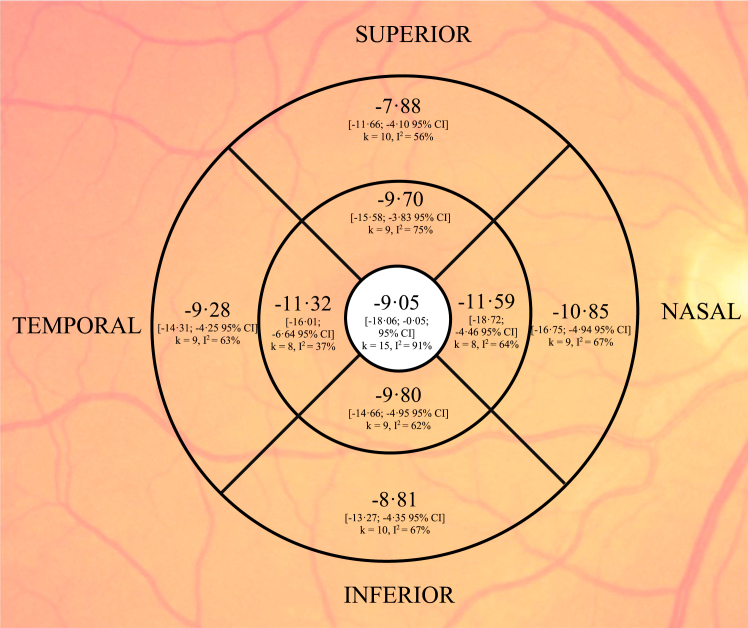
Meta-analysis of right eye of macular thickness differences (μm) between schizophrenia spectrum disorder cases and controls according to the ETDRS (Early Treatment of Diabetic Retinopathy Study) subfields.

More studies were available to assess the ETDRS central foveal thickness subfield, which demonstrated significant thinning in SSD cases and significant heterogeneity (−9.05 μm, 95% CI, −18.06 to −0.05, *I*^2^ = 91%, k = 15). Heterogeneity analysis identified Sarkar *et al.* (41) (composed of acute episode cases) as a significant outlier/influential study. Exclusion of this study improved heterogeneity while continuing to show mean thinning in SSD cases (−6.22 μm, 95% CI, −11.21 to −1.22, *I*^2^ = 53%, k = 14). All other ETDRS subfields showed significant pooled thinning with moderate to high heterogeneity (Figure 6).

### Inner Retinal Macular Submeasures

Pooled analysis of the macular GCIPL thickness demonstrated that SSD cases had a significant thinning of −2.44 μm (95% CI, −4.13 to −0.76, *I*^2^ = 30%, k = 8). No significant outliers were detected. Visual inspection of funnel plots suggests that 1 study (42) may reflect publication bias. There was an insufficient number of studies (k = 2) for meta-analysis of the macular retinal nerve fiber layer, and these studies did not detect significant differences between cases and controls using the automated device algorithms (43,44). Manual measurements of single macular B scan slices in the temporal, foveal, and nasal regions have demonstrated statistically significant thinning of the macular GCPL (45,46); however, this method has yet to be validated.

### Acute Episode and Stable SSD Subgroups

A meta-analysis of global pRNFL and average macular thickness comparing acute episode (k = 8, *n*_SSD_ = 235, *n*_control_ = 179) and stable SSD cohorts (k = 18, *n*_SSD_ = 669, *n*_control_ = 559) was undertaken. The mean reported disease duration was 2.65 ± 4.96 (*n* = 156, k = 5) and 13.6 ± 10.0 (*n* = 427, k = 11) years for the acute and stable SSD subgroups, respectively. Significant reductions in global pRNFL (−3.47 μm, 95% CI, −5.17 to −1.77, *I*^2^ = 42%, k = 15) and average macular thickness (−7.99 μm, 95% CI, −12.70 to −3.30, *I*^2^ = 47%, k = 8) was demonstrated in stable SSD while differences in global pRNFL (−0.36 μm, 95% CI, −2.63 to +1.92, *I*^2^ = 0%, k = 7) and average macular thickness (−5.82 μm, 95% CI, −24.00 to +12.35, *I*^2^ = 82%, k = 4) in acute SSD cases failed to reach statistical significance. No significant changes were found after exclusion of outliers/influential studies.

### OCT Measures in Relation to Other Clinical Disease Markers

Participants with treatment-resistant schizophrenia were reported to have thinner choroid, ganglion cell, and inner plexiform volumes than treatment-responsive cases (47,48). Choroidal atrophy was greater in clozapine and first-generation antipsychotics than second-generation antipsychotics; however, clozapine was exclusively used in treatment-resistant cases (48). No significant differences were found for pRNFL and inner plexiform volumes between those treated with clozapine, first-generation, or second-generation antipsychotics (48).

Patients with schizophrenia who were without insight had significantly thinner macular GCIPL and pRNFL values than cases with preserved insight (49). Patients with schizophrenia who were presenting with their first episode of auditory and visual hallucinations with specific magnetic resonance imaging characteristics (structural gray matter volume reduction and increased global functional connectivity density) were associated with pRNFL and macular thickness atrophy compared with control participants (46). Furthermore, these cases were found to have statistically significant progression of atrophy after 6 and 36 months of antipsychotic treatment (while no progression occurred in controls) (50,51).

Cases with auditory hallucinations exhibited temporal pRNFL, nasal pRNFL, and macular atrophy; however, the severity of auditory or visual hallucinations could not determine the severity of atrophy (46,52). In a separate study, a modified score using only the visual items from the Bonn Scale for the Assessment of Basic Symptoms were found to be correlated with macular thickness atrophy (53). There have been other reports of statistically significant but weak positive correlations in macular GCIPL thickness with clinical global impression ratings (47); pRNFL thickness with components of cognitive functional testing (visuospatial/constructional, immediate memory, language, word/color processing) (37,54), serum ciliary neurotrophic factor (37); and specific choroidal thickness sectors with disease duration (39). Moderate significant correlations were reported in temporal parafoveal total retinal/ganglion cell complex thickness and foveal total retinal/photoreceptor complex with contrast sensitivity at 0.5 cycles per degree of visual angle. Studies of choroidal thickness differences in schizophrenia have shown mixed results (48,55,56).

### OCT Angiography Studies

Due to poor interdevice correlation and limited number of studies, a meta-analysis was not appropriate. Individual studies that included chronic and short-term SSD cases have found less temporal peripapillary vascular density (57), total radial peripapillary vascular density (39), lower deep macular complex vessel density (39), lower macular perfusion density (58), and a larger foveal avascular zone (58). These studies did not report smoking status. Bannai *et al.* (59) found a subset of schizophrenia cases with disease durations of <5 years who had significantly higher rates of smoking and exhibited right eye–only increased superficial retinal and choriocapillaris vascular densities. Macular OCT angiography (OCT-A) measurements did not reach statistical significance when correlated with chlorpromazine equivalent dosing, age at first hospitalization, and self-reported disease duration (58).

## Discussion

This systematic review and meta-analysis found significant associations with SD-OCT pRNFL and total macular and inner macular GCIPL thinning in SSD.

The association of pRNFL atrophy in SSD was robust, even after exclusion of metabolic disease and low-quality, outlier/influential studies. SSD age was correlated with pRNFL atrophy, suggesting increased rates of retinal neurodegeneration over time. However, smoking, BMI, and disease factors (duration, PANSS scores, chlorpromazine equivalent antipsychotic dose) were either not significantly associated with retinal thinning or lacked enough studies for subanalysis. Only a single study (60) consisting of chronic stable SSD cases was able to control for all major confounders in this review and could only demonstrate macular inner plexiform layer thinning but not pRNFL or other macular OCT parameters. Additional studies are needed with careful control of these covariates to detect potential subtle thinning of other parameters in SSD cases.

Diffuse inner retinal atrophy would result in maximal pRNFL thinning at the inferior quadrant because it anatomically receives the largest contribution of retinal nerve fibers; however, the degree of inferior-superior asymmetry in the normal optic nerve is approximately 11% (61) while we found 46% more inferior versus superior thinning. Five of the 6 individual studies (37,38,47,49,62) that demonstrated inferior pRNFL atrophy utilized eye examinations to rule out ocular causes of focal retinal insult. Publication bias appears to have played a role in superior and inferior pRNFL outcomes; however, significant asymmetry persisted even after statistical correction. Evidence for selective retinal degeneration in neurological disease states has been found in Alzheimer’s dementia, Parkinson’s disease, Huntington’s disease, and demyelinating optic neuritis (63). In schizophrenia, a limited number of studies (64,65) have shown reduced white matter reductions within the temporal lobe, which contains the inferior fibers of the visual pathway, thereby providing a neuroanatomical basis for this observation. However, the exact mechanisms responsible for highly selective inferior pRNFL atrophy in SSD remain unclear.

Continental European, Asian, Cirrus OCT, outpatients, and studies incorporating eye examinations found statistically significant pRNFL atrophy in SSD. These groups were characterized by k ≥ 10 studies with the exception of continental Asian studies (k = 4), which demonstrated more severe pRNFL (37,46), average macular (38), and central foveal thickness atrophy (41). This Asian subgroup had a greater proportion of inpatients (37,38), acute episodes (37,38,41,46), and smokers in the SSD group (37). We hypothesize that this Asian subgroup represents a highly neurodegenerative phenotype, with the cultural perceptions of mental illness (66) and access to care (67) in many Asian societies resulting in a selection bias of the most severe cases and extended periods of untreated disease. Patient history cannot be relied upon to identify the presence of asymptomatic ophthalmic disease [such as glaucoma (68) and diabetic retinopathy (69)], which are more prevalent in schizophrenia. Our analysis found that only studies with ophthalmic screening protocols could demonstrate significant thinning, demonstrating the need for ophthalmic screening with a minimum of visual acuity, intraocular pressure, and anterior and dilated posterior segment examination prior to OCT assessment.

Silverstein (70) has suggested that antipsychotic dopamine receptor blockade results in inner retinal atrophy based on the observation that all retinal cell types are responsive to dopamine, and ganglion cell atrophy has been observed in Parkinson’s disease. The mechanism is hypothesized to arise from diminished dopamine input to ganglion cells (71). Furthermore, other drug-related sources of inner retinal atrophy include drug-induced retinopathy (72) and antipsychotic-induced diabetic retinopathy. The average reported chlorpromazine equivalent dose in this meta-analysis was well below worldwide prescribing levels (73); however, individual cohorts were at the upper end of dosing levels, and yet we were unable to demonstrate a dose-dependent relationship with retinal atrophy in our statistical models.

We also explored whether acute episodes were indicative of neuroinflammation (correlated with retinal thickening) or as a marker of severe neurodegeneration (correlated with retinal thinning). Our analysis failed to detect significant differences in acute episode SSD cases while stable SSD cases demonstrated significant pRNFL and macular atrophy. Attributing the much shorter disease duration in acute episode SSD analysis may be enough to explain this finding because stable SSD cases simply might have had more time for neurodegeneration to occur. However, multiple hypotheses emerged from the lack of detectable changes in acute episode SSD including 1) masking of atrophy through neuroinflammation, 2) failure to detect early onset neurodegeneration, and 3) a null hypothesis. The first postulation of acute neuroinflammation masking detectable atrophy arose from Ascaso’s (74) relatively small (*n* = 10) cohort; however, low statistical power is more likely. The only longitudinal study of acute SSD comes from Zhuo’s cohort (50,51) which found retinal atrophy at baseline, accelerated loss at 6 and 36 months, and stabilizing at 3.5 years of treatment. This raises an interesting question about whether antipsychotic treatment (except for proinflammatory clozapine) may be reducing neuroinflammation to reveal underlying atrophy. Only a single SD-OCT study compared acute and stable SSD groups of similar disease durations: central foveal thickness and 2 pRNFL sectors were found to be thinner in acute SSD cases; however, this could not be replicated in both eyes (75). The lack of retinal thickening and negative findings from our analysis do not actively support neuroinflammation, and if present, it cannot be differentiated from longitudinal neurodegeneration.

Neurovascular dysfunction occurs in schizophrenia (76) which is supported by brain hypoperfusion in neuroimaging studies (77,78) and a number of neurovascular genetic associations (79). Our review of OCT-A findings suggests that SSD cases exhibit reduced retinal perfusion and vessel density at the macular and optic disc. This may reflect microangiographic destruction from neuroinflammation (76); however, numerous retinal vasculopathies exists in SSD including diabetes, hypertension, and smoking. The contradictory findings from Bannai *et al.* (59) of increased choriocapillaris and superficial retinal vessel density may be explained by the statistically significant number of smokers within the SSD group. Cigarette smoking has variable effects on retinal vascular densities as measured on OCT-A (80,81). The well-known retinal venous vasodilatory effects of chronic smoking may confer a degree of vascular congestion at the choriocapillaris and superficial plexus, where these capillary networks are in close proximity to large draining venules (81). In addition, retinal thinning in SSD permits further OCT signal penetration and subsequently enhances choroidal vessel density images (82). Nevertheless, the alternative hypothesis of acute neuroinflammation driving microvascular structural congestion remains a viable hypothesis that needs to be disentangled from these confounding issues. Furthermore, OCT-A algorithms are not standardized between devices; therefore, current and future findings would benefit from replication studies across different OCT-A devices.

This meta-analysis focused on Fourier domain OCT devices, which offer better sensitivity to subtle retinal structural changes and allow segmentation of individual retinal layers compared with older devices (83). Our results are consistent with previous meta-analyses incorporating older TD-OCT data which include pRNFL sectors (19, 20, 21, 22, 23), macular GCIPL (20,22), average macular thickness (22,23), and macular regional ETDRS sectors (22). The two most recent meta-analyses differed significantly in methodology including averaging bilateral eye summary data (22), fixed-effects modeling (22) (which is more likely to produce statistical significance), and restrictions on study language (22,23). The meta-analysis by Gonzalez-Diaz *et al.* (23) was the most recent one and did not detect significant retinal atrophy with TD-OCT data while SD-OCT detected significant thinning. In addition, they had found a greater mean degree of global pRNFL and macular volume (but not total thickness) in right eyes; however, this was not statistically significant (23). Because right eyes are typically measured by convention, we preferred to extract data from right eye measurements if bilateral data were reported to reduce the risk of violating statistical assumptions related to pooling correlated data. Previous pooled meta-analyses failed to demonstrate statistically significant atrophy in all pRNFL and macular OCT subgrid measures [except for Gonzalez-Diaz *et al.* (23)], but we demonstrated significance in all measures. These findings persisted even after being challenged with sensitivity, influence, outlier, subgroup, multivariate regression, and publication bias analyses.

There are several limitations to consider. The first is that there is a lack of studies reporting important confounders influencing retinal findings (such as smoking and acute relapse). Second, SSDs represent a heterogeneous group, as demonstrated in our analysis showing that schizophrenia cases had 20% more pRNFL atrophy than SSDs combined. Even schizophrenia itself is characterized by several possible disease states with relapse, remission, distinctive phenotypes with positive/negative symptoms, and cognitive impairment. Thirdly, knowledge of the trajectory and natural history of retinal thinning in SSD is needed to assess the diagnostic and prognostic value of OCT in SSD. In particular, the question of whether an inherited subnormal retinal thickness represents a neurodevelopmental trait that proceeds development of clinical SSD. This hypothesis is supported by the observation of thinner IPL sublayers in first-degree relatives of probands with schizophrenia (60). The correlation with age and increasing pRNFL atrophy in SSD in our analysis supports the neurodegenerative hypothesis of SSD; however, we do not have data to refute a neurodevelopmental hypothesis. A high-quality, longitudinal, prospective case-control study is needed to address these limitations.

The current clinical diagnostic categories of SSDs lack the precision to correlate against the numerous contending neurobiological proxies for neurodegeneration and neuroinflammation (84). However, we believe that OCT technology has the potential to identify highly neurodegenerative phenotypes, detect neurodevelopmental retinal abnormalities that precede clinical diagnosis of SSD, and as a therapeutic biomarker for neurodegeneration and neuroinflammation.

### Conclusions

SSDs are associated with peripapillary retinal nerve fiber, macular ganglion cell-inner plexiform sublayer, and total macular atrophy. There are a limited number of studies that have controlled for all major confounders that influence retinal findings. Future prospective longitudinal studies that control for smoking and cardiometabolic and ocular disease and assessment during acute and chronic disease states can assist in validating retinal OCT, alongside neuroimaging and electrophysiology, as a useful biomarker in diagnostic and therapeutic monitoring.

## Supplementary Material

Supplementary Data

Key Resources Table
