# Supplementary material for: Optical Coherence Tomography in Schizophrenia Spectrum Disorders: A Systematic Review and Meta-analysis
Source: Biol Psychiatry Glob Open Sci. 2023 Aug 30;4(1):19–30. doi: 10.1016/j.bpsgos.2023.08.013 (PMC10654004; doi:10.1016/j.bpsgos.2023.08.013)
Supplement: Key Resources Table [file mmc2.pdf]

SUPPLEMENTARY INFORMATION

Optical Coherence Tomography in Schizophrenia Spectrum Disorders – A Systematic Review and Meta-analysis

Shew *et al.*

PRISMA Checklist

Section and Topic	Item #	Checklist item	Location where item is reported
**TITLE**			
Title	1	Identify the report as a systematic review.	Page 1
**ABSTRACT**			
Abstract	2	See the PRISMA 2020 for Abstracts checklist.	Page 2
**INTRODUCTION**			
Rationale	3	Describe the rationale for the review in the context of existing knowledge.	Page 6-7
Objectives	4	Provide an explicit statement of the objective(s) or question(s) the review addresses.	Page 7
**METHODS**			
Eligibility criteria	5	Specify the inclusion and exclusion criteria for the review and how studies were grouped for the syntheses.	Page 7-8
Information sources	6	Specify all databases, registers, websites, organisations, reference lists and other sources searched or consulted to identify studies. Specify the date when each source was last searched or consulted.	Page 8
Search strategy	7	Present the full search strategies for all databases, registers and websites, including any filters and limits used.	Page 8, Supplementary
Selection process	8	Specify the methods used to decide whether a study met the inclusion criteria of the review, including how many reviewers screened each record and each report retrieved, whether they worked independently, and if applicable, details of automation tools used in the process.	Page 8
Data collection process	9	Specify the methods used to collect data from reports, including how many reviewers collected data from each report, whether they worked independently, any processes for obtaining or confirming data from study investigators, and if applicable, details of automation tools used in the process.	Page 8-9
Data items	10a	List and define all outcomes for which data were sought. Specify whether all results that were compatible with each outcome domain in each study were sought (e.g. for all measures, time points, analyses), and if not, the methods used to decide which results to collect.	Page 8, Supplementary
	10b	List and define all other variables for which data were sought (e.g. participant and intervention characteristics, funding sources). Describe any assumptions made about any missing or unclear information.	Page 8
Study risk of bias assessment	11	Specify the methods used to assess risk of bias in the included studies, including details of the tool(s) used, how many reviewers assessed each study and whether they worked independently, and if applicable, details of automation tools used in the process.	Page 9, Supplementary
Effect measures	12	Specify for each outcome the effect measure(s) (e.g. risk ratio, mean difference) used in the synthesis or presentation of results.	Page 9
Synthesis methods	13a	Describe the processes used to decide which studies were eligible for each synthesis (e.g. tabulating the study intervention characteristics and comparing against the planned groups for each synthesis (item #5)).	Supplementary
	13b	Describe any methods required to prepare the data for presentation or synthesis, such as handling of missing summary statistics, or data conversions.	Page 9
	13c	Describe any methods used to tabulate or visually display results of individual studies and syntheses.	Page 9-10
	13d	Describe any methods used to synthesize results and provide a rationale for the choice(s). If meta-analysis was performed, describe the model(s), method(s) to identify the presence and extent of statistical heterogeneity, and software package(s) used.	Page 9
	13e	Describe any methods used to explore possible causes of heterogeneity among study results (e.g. subgroup analysis, meta-regression).	Page 9-10

Section and Topic	Item #	Checklist item	Location where item is reported
	13f	Describe any sensitivity analyses conducted to assess robustness of the synthesized results.	Page 10
Reporting bias assessment	14	Describe any methods used to assess risk of bias due to missing results in a synthesis (arising from reporting biases).	Page 10
Certainty assessment	15	Describe any methods used to assess certainty (or confidence) in the body of evidence for an outcome.	Page 9-10
**RESULTS**			
Study selection	16a	Describe the results of the search and selection process, from the number of records identified in the search to the number of studies included in the review, ideally using a flow diagram.	Figure 1
	16b	Cite studies that might appear to meet the inclusion criteria, but which were excluded, and explain why they were excluded.	Figure 1, Supplementary
Study characteristics	17	Cite each included study and present its characteristics.	Page 10, Supplementary
Risk of bias in studies	18	Present assessments of risk of bias for each included study.	Supplementary
Results of individual studies	19	For all outcomes, present, for each study: (a) summary statistics for each group (where appropriate) and (b) an effect estimate and its precision (e.g. confidence/credible interval), ideally using structured tables or plots.	Figure 2,3 Supplementary
Results of syntheses	20a	For each synthesis, briefly summarise the characteristics and risk of bias among contributing studies.	Supplementary
	20b	Present results of all statistical syntheses conducted. If meta-analysis was done, present for each the summary estimate and its precision (e.g. confidence/credible interval) and measures of statistical heterogeneity. If comparing groups, describe the direction of the effect.	Page 11-16 Figure 2,3 Supplementary
	20c	Present results of all investigations of possible causes of heterogeneity among study results.	Page 11-16 Figure 2,3 Supplementary
	20d	Present results of all sensitivity analyses conducted to assess the robustness of the synthesized results.	Table 1 Supplementary
Reporting biases	21	Present assessments of risk of bias due to missing results (arising from reporting biases) for each synthesis assessed.	Page 12-16 Figure 6 Supplementary
Certainty of evidence	22	Present assessments of certainty (or confidence) in the body of evidence for each outcome assessed.	Page 11-16 Figure 2,3 Supplementary
**DISCUSSION**			
Discussion	23a	Provide a general interpretation of the results in the context of other evidence.	Page 18-23
	23b	Discuss any limitations of the evidence included in the review.	Page 24-25
	23c	Discuss any limitations of the review processes used.	Page 24-25
	23d	Discuss implications of the results for practice, policy, and future research.	Page 18, 20, 23, 25
**OTHER INFORMATION**			
Registration and protocol	24a	Provide registration information for the review, including register name and registration number, or state that the review was not registered.	Page 7
	24b	Indicate where the review protocol can be accessed, or state that a protocol was not prepared.	Page 7

Section and Topic	Item #	Checklist item	Location where item is reported
	24c	Describe and explain any amendments to information provided at registration or in the protocol.	NA
Support	25	Describe sources of financial or non-financial support for the review, and the role of the funders or sponsors in the review.	Page 25
Competing interests	26	Declare any competing interests of review authors.	Page 25
Availability of data, code and other materials	27	Report which of the following are publicly available and where they can be found: template data collection forms; data extracted from included studies; data used for all analyses; analytic code; any other materials used in the review.	Supplementary

Extraction Data Items

**Study Design**
Author
Date published
Study period
Country
Setting
Diagnostic criteria
Inclusion and exclusion criteria
Eye examination
Optical coherence tomography device
**Population**
Demographics (number, age, sex)
Medication dose (chlorpromazine equivalent)
Smoking status
Cardiometabolic disease status
BMI
**Comparators and Exposure**
Illness duration
Illness severity (PANSS)
**Outcomes**
Peripapillary retinal nerve fibre layer thickness (pRNFL)
Macular retinal nerve fibre layer volume (mRNFL)
Macular ganglion cell – inner plexiform layer volume (GCIPL)
Macular Early Treatment of Diabetic Retinopathy Study (ETDRS) Grid thickness

Study data items extracted for analysis.

Search Strategy

Medline

MESH Search Strategy

1 exp "schizophrenia spectrum and other psychotic disorders"/ 158446

2 Tomography, Optical Coherence/ 43369

3 exp Retina/ 148080

4 1 and (2 or 3) 141

Comprehensive Search Strategy

((schizophrenia or schizoaffective or psychosis or psychiatry or psychiatric) and ("optical coherence tomography" or "ganglion cell" or retina*)).ti. or ((schizophrenia or schizoaffective or psychosis or psychiatry or psychiatric) and ("optical coherence tomography" or "ganglion cell" or retina*)).ab. or ((schizophrenia or schizoaffective or psychosis or psychiatry or psychiatric) and ("optical coherence tomography" or "ganglion cell" or retina*)).kf. 438

Pubmed

Search: (schizophrenia[Title/Abstract] OR schizoaffective[Title/Abstract] OR psychosis[Title/Abstract] OR psychiatry[Title/Abstract] OR psychiatric[Title/Abstract]) AND ("optical coherence tomography"[Title/Abstract] OR "ganglion cell"[Title/Abstract] OR retina*[Title/Abstract]) 465

EMBASE (1980 to current)

MESH Search Strategy

1 exp schizophrenia spectrum disorder/ 189620

2 exp retina/ 135981

3 exp optical coherence tomography/ 80328

4 1 and (3 or 3) 130

Search: ((schizophrenia or schizoaffective or psychosis or psychiatry or psychiatric) and ("optical coherence tomography" or "ganglion cell" or retina*)).ti. or ((schizophrenia or schizoaffective or psychosis or psychiatry or psychiatric) and ("optical coherence tomography" or "ganglion cell" or retina*)).ab. or ((schizophrenia or schizoaffective or psychosis or psychiatry or psychiatric) and ("optical coherence tomography" or "ganglion cell" or retina*)).kf. 686

Scopus

Search: TITLE-ABS-KEY ((schizophrenia OR schizoaffective OR psychosis OR psychiatry OR psychiatric) AND ("optical coherence tomography" OR "ganglion cell" OR retina*)) 1,362

PsycInfo (1806 to current)

MESH Search Strategy

APA PsycInfo <1806 to May Week 4 2022>

1 exp psychosis/ 123902
2 exp Retina/ 8960
3 1 and 2 68

((schizophrenia or schizoaffective or psychosis or psychiatry or psychiatric) and ("optical coherence tomography" or "ganglion cell" or retina*)).ti. or ((schizophrenia or schizoaffective or psychosis or psychiatry or psychiatric) and ("optical coherence tomography" or "ganglion cell" or retina*)).ab. or ((schizophrenia or schizoaffective or psychosis or psychiatry or psychiatric) and ("optical coherence tomography" or "ganglion cell" or retina*)).cw. 207

PSYNDEX

Search: (schizophrenia OR schizoaffective OR psychosis OR psychiatry OR psychiatric) AND ("optical coherence tomography" OR "ganglion cell" OR retina*) 16

WHO International clinical trials registry platform

Search: (schizophrenia OR schizoaffective OR psychosis OR psychiatry OR psychiatric) AND ("optical coherence tomography" OR "ganglion cell" OR retina*) With results only 3

Cochrane Controlled Register of Trials

Search: ((schizophrenia OR schizoaffective OR psychosis OR psychiatry OR psychiatric) AND ("optical coherence tomography" OR "ganglion cell" OR retina*).):ti,ab,kw (Word variations have been searched) 37

Table S1. Study Inclusion and Exclusion Criteria

Study	Inclusion Criteria	Exclusion Criteria
**Qualitative Analysis**		
Alizadeh[1]	18 to 65 years old<br>Males only<br>Stable antipsychotic medication at therapeutic dosages<br>BMI 18-28	Ocular pathology, previous surgery, refractive error >4 dioptres<br>Neurological, metabolic or endocrine pathology<br>Unstable mental state<br>Substance abuse (Except smoking)
Altun[2]	18 to 65 years old<br>On risperidone or clozapine for >6mo	Ocular pathology, trauma/surgery, refractive error >2 dioptres<br>Neurological, metabolic, or endocrine pathology<br>Comorbid psychiatric disease<br>Chlorpromazine equivalent >300mg
Asanad[3]	Stable mental state	Ocular pathology, previous surgery (except for uncomplicated cataract surgery)<br>Neurological pathology<br>Substance abuse (last month) or dependence (last three months) (Except smoking and marijuana use)
Bozali[4]		Ocular pathology, refractive error >3 dioptres, intraocular pressure >21mmHg<br>Metabolic or endocrine pathology
Celik[5]	18 to 65 years old<br>SZ Treatment resistant: Minimal/no response to 2 or more antipsychotic trials in therapeutic range of at least 6 weeks.<br>SZ Treatment responsive: Stable disease at baseline visit where medication change/increase was not considered	Ocular pathology, refractive error >1 dioptres<br>Cardio
Delibas[6]	18 to 65 years old<br>No relapse in last 6/12	Ocular pathology, trauma/surgery<br>Neurological, metabolic, or endocrine pathology<br>Substance abuse/dependence (Except smoking)
Gandu[7]	18-60 years old<br>(Bipolar and Schizophrenia Network on Intermediate Phenotype-2 Criteria)	Ocular pathology, trauma, refractive error >4 dioptres<br>Neurological pathology<br>Substance abuse/dependence (Except smoking)
Hosak[8]	18-65 years old	Comorbid psychiatric disease<br>Hypertension, heart disease or stroke and disorders potentially influencing retinal microvasculature<br>Ocular pathology
Jerotic[9]	>18 years old<br><15 years duration in remission<br>Intelligence quotient >80	Ocular pathology, refractive error >2 dioptres<br>Neurological, metabolic, or endocrine pathology<br>Substance abuse/dependence (Except smoking)
Kaya[10]	18-50<br>Able to read and write<br>Stable SZ: No acute psychotic exacerbation, no requirement for change of antipsychotic medication in the past month, no history of hospitalization in the previous 12 months	Ocular pathology<br>Neurological pathology<br>Substance abuse/dependence (Except smoking)
Koman-Wierdak[11]	SZ: In last week of acute remission	Ocular pathology<br>Metabolic, or endocrine pathology<br>Substance abuse/dependence (Except smoking)
Kozub[12]		Ocular pathology, High myopia with retinal changes
Kurt[13]	18-64 years old	Ocular pathology, refractive error >2 dioptres<br>Neurological, metabolic, or endocrine pathology<br>Substance abuse/dependence (Except smoking)
Kurtulmus[14]	23-65 years old<br>SZ with no admissions in last 6 months or changes in last 2 months to medications	Ocular pathology, retinal surgery, refractive error ≥3 dioptres<br>Neurological pathology<br>Comorbid psychiatric disease or intellectual impairment
Lai[15]	18-65 years old<br>First episode SZ + SA group: No more than 1 prior hospitalization, with the hospitalization occurring within the past two years<br>Chronic SZ + SA: At least two prior hospitalizations<br>Controls: <30yrs and >30yrs old<br>Understands English	Ocular pathology, trauma, abnormal corrected visual acuity<br>Diabetes or high blood pressure<br>Neurological, intellectual, developmental disorders<br>Head trauma with loss of consciousness >10min<br>Comorbid psychiatric disease, electroconvulsive therapy within last 8 weeks
Lee[16]	>18 years old<br>Acute SZ: Inpatient, duration <2yrs<br>Chronic SZ: Outpatient, duration 2-10yrs<br>Long Term Chronic SZ: Outpatient, duration >10yrs	Ocular pathology, trauma/surgery, refractive error >2 dioptres<br>Neurological, metabolic, or endocrine pathology
Liu[17]	18 to 65 years old	Ocular pathology, refractive error >4 dioptres<br>Neurological, metabolic or endocrine pathology<br>Substance abuse/dependence (Except smoking)
Miller[18]	18 to 68 years old<br>Treated with any antipsychotic except clozapine for at least 8 weeks or antipsychotic naive	Ocular pathology<br>Neurological pathology, unstable medical illness, pregnant or nursing, anaemia, renal insufficiency

	Willing to participate in CBT<br>Sufficient proficiency in English to complete assessments<br>Score of at least 3 on the SAPS at two assessments, four weeks apart<br>(D-Cycloserine Augmentation of Cognitive Behavioural Therapy for Delusions criteria)	SSRI or clozapine treatment, active alcohol or other substance abuse within six weeks
Mota[19]	>18 years old<br>SZ: Clinically stable disease	Ocular pathology, refractive error >6 dioptres<br>Neurological, metabolic, or endocrine pathology<br>Comorbid psychiatric disease<br>Substance abuse/dependence (Except smoking)
Sarkar[20]	18 to 50 years old<br>Acute SZ: Symptoms <3 months, duration <5 years	Ocular pathology, surgery/trauma, refractive error >2 dioptres<br>Neurological, metabolic or endocrine pathology<br>Chlorpromazine, Thioridazine therapy
Schönfeldt-Lecuona[21]	18-65 years old	Ocular pathology, recent surgery, refractive error >6 dioptres, intraocular pressure >21mmHg<br>Neurological, metabolic or endocrine pathology<br>Comorbid psychiatric disorder<br>Substance abuse (Except smoking)<br>High-dose steroid therapy
Silverstein[22]	21 to 60 years old	Ocular pathology, trauma<br>Neurological pathology<br>Comorbid psychiatric disease<br>Substance abuse/dependence (Except smoking)
Topcu-Yilmaz[23]	SZ: Admitted, partially stable enough for ophthalmic exam	Ocular pathology, trauma/surgery, refractive error >2 dioptres, intraocular pressure >21mmHg<br>Neurological, metabolic, or endocrine pathology
Yilmaz[24]		Ocular pathology, surgery<br>Metabolic pathology<br>Smokers
**Qualitative Analysis**		
Bannai[25]		Ocular pathology, refractive error >4 dioptres<br>Neurological or endocrine pathology<br>Intellectual disability<br>Current pregnancy or breast feeding<br>Substance abuse/dependence within the past 6 months (Except smoking)
Budakoglu[26]	>18 years old<br>SZ: Stable disease with >2 years duration	Ocular pathology, trauma/surgery, refractive error >1 dioptres, intraocular pressure >21mmHg
Huang[27]	18-35 years old<br>SZ: First untreated (>3 weeks of no antipsychotic) episode, insight to disease symptoms, ability to complete interview fluently	Ocular pathology, myopia >6.0D<br>Neurological, metabolic, endocrine, hepatic, renal or other chronic pathology<br>Intelligence quotient <80<br>Substance abuse<br>Electroconvulsive therapy
Jerotic[28]	18-40 years old<br><15 years duration in remission	Ocular pathology, refractive error >2 dioptres<br>Neurological, metabolic, or endocrine pathology<br>Substance abuse/dependence (Except smoking)
Liu[29]	18 to 60 years old<br>SZ: Antipsychotic therapy for >3 months	Ocular pathology, refractive error >2 dioptres<br>Neurological, metabolic or endocrine pathology, head trauma<br>Substance abuse/dependence (Except smoking)
Orum[30]	SZ on same class antipsychotic for >2 years, duration >5yrs, in remission	Ocular pathology, surgery/trauma, refractive error >1 dioptres<br>Neurological, metabolic or endocrine pathology<br>Comorbid psychiatric disease<br>Substance abuse/dependence (Except smoking)
Samani[31]		Ocular pathology, surgery/trauma, refractive error >6 dioptres<br>Metabolic or endocrine pathology<br>Substance abuse/dependence (Except smoking)
Silverstein[32]	18-65 years old<br>English speaker	Ocular pathology, abnormal visual acuity<br>Neurological, metabolic or endocrine pathology<br>Electroconvulsive therapy in the last 8 weeks
Zhuo[33]	18 to 30 years old<br>SZ: First episode of psychotic symptoms in a mental health hospital. Both visual and auditory hallucinations. No antipsychotic medication taken for ≥3 weeks prior to study.	Ocular pathology, severe myopia<br>Neurological, metabolic, endocrine, hepato-biliary, renal respiratory, cardiovascular pathology<br>Intelligence quotient <80<br>Loss of consciousness >5min<br>Electroconvulsive therapy<br>Left-handedness<br>Substance abuse<br>MRI contradiction
Zhuo[34]	18 to 30 years old<br>SZ: First episode of psychotic symptoms in a mental health hospital. Visual disturbances as defined by in the	Ocular pathology, severe myopia<br>Neurological, metabolic, endocrine, hepato-biliary, renal respiratory, cardiovascular pathology<br>Intelligence quotient <80

	Bonn Scale for Assessment of Basic Symptoms. Auditory hallucinations as defined by the auditory hallucination rating scale. No antipsychotic medication taken for ≥3 weeks prior to study.	Loss of consciousness >5min<br>Electroconvulsive therapy<br>Left-handedness<br>Substance abuse<br>MRI contradiction
Zhuo[35]	18 to 30 years old<br>SZ: First episode of psychotic symptoms in a mental health hospital. Both visual and auditory hallucinations. No antipsychotic medication taken for ≥3 weeks prior to study.	Ocular pathology, severe myopia<br>Neurological, metabolic, endocrine, hepato-biliary, renal respiratory, cardiovascular pathology<br>Intelligence quotient <80<br>Loss of consciousness >5min<br>Electroconvulsive therapy<br>Left-handedness<br>Substance abuse<br>MRI contradiction
Zhuo[36]	18 to 30 years old<br>SZ: First episode of psychotic symptoms in a mental health hospital. Visual disturbances as defined by in the Bonn Scale for Assessment of Basic Symptoms (Score >16). No antipsychotic medication taken for ≥3 weeks prior to study.	Ocular pathology, severe myopia<br>Neurological, metabolic, endocrine, hepato-biliary, renal respiratory, cardiovascular pathology<br>Intelligence quotient <80<br>Loss of consciousness >5min<br>Electroconvulsive therapy<br>Left-handedness<br>Substance abuse<br>MRI contradiction

Table S2. Study Quality Assessment - Newcastle-Ottawa Scale

Quantitative Analysis										
**Study**	**Sel 1**	**Sel 2**	**Sel 3**	**Sel 4**	**Com 1 (Ocular pathology)**	**Com2 (Cardio-metabolic disease)**	**Exp 1**	**Exp 2**	**Exp 3**	**Total**
Alizadeh [1]	X	-	X	X	X	X	X	X	-	**7**
Altun [2]	X	-	X	X	X	X	X	-	-	**6**
Asanad [3]	X	-	X	X	X	-	X	X	X	**7**
Bozali[4]	X	-	-	-	X	X	X	-	-	**4**
Celik [5]	X	-	-	X	X	X	X	-	-	**5**
Delibas [6]	X	-	-	X	X	X	X	X	-	**6**
Gandu [7]	X	-	X	X	X	-	X	X	-	**6**
Hosak[8]	X	-	-	X	X	X	X	-	-	**5**
Jerotic [9]	X	-	-	X	X	X	X	-	-	**5**
Kaya[10]	X	-	X	X	X	X	X	X	-	**7**
Koman-Wierdak[11]	X	-	-	X	X	X	X	X	-	**6**
Kozub[12]	-	X	X	-	X	-	-	-	-	**3**
Kurt[13]	X	X	-	X	X	-	X	-	-	**5**
Kurtulmus[14]	X	-	X	X	X	X	X	X	-	**7**
Lai[15]	X	X	X	X	X	X	X	-	-	**7**
Lee[16]	X	X	X	X	X	X	X	X	-	**8**
Liu[17]	X	-	X	X	X	X	X	X	-	**7**
Miller[18]	X	-	-	-	X	-	X	-	-	**3**
Mota[19]	X	-	X	-	X	X	X	-	-	**5**
Sarkar[20]	X	-	X	X	X	X	X	-	X	**7**
Schönfeldt-Lecuona[21]	X	-	-	X	X	X	X	X	-	**6**
Silverstein[22]	X	-	X	X	X	X	X	-	-	**6**
Topcu-Yilmaz[23]	X	-	-	X	X	X	X	-	-	**5**
Yilmaz[24]	-	-	X	-	X	X	-	-	-	**3**
Qualitative Analysis										
**Study**	**Sel 1**	**Sel 2**	**Sel 3**	**Sel 4**	**Com 1**	**Com2**	**Exp 1**	**Exp 2**	**Exp 3**	**Total**
Bannai[25]	X	-	-	X	X	X	X	-	X	**6**
Budakoglu[26]	X	-	X	-	X	X	X	-	-	**5**
Huang[27]	X	-	-	X	X	X	X	-	-	**5**
Jerotic[28]	X	-	X	X	X	X	X	-	-	**6**
Liu[29]	X	-	-	X	X	X	X	X	-	**6**
Orum[30]	X	-	-	X	X	X	X	-	-	**5**
Samani[31]	X	X	-	X	X	X	X	-	-	**6**
Silverstein[32]	-	-	-	X	X	X	-	-	-	**3**
Zhuo[33]	X	-	-	X	X	X	X	X	-	**6**

Zhuo[34]	X	X	-	X	X	X	X	X	-	**7**
Zhuo[35]	X	X	-	X	X	X	X	X	-	**7**
Zhuo[36]	X	X	-	X	X	X	X	X	-	**7**

Table S3. Summary of Study Characteristics

Quantitative Analysis									
**Study**	**Period**	**Country**	**Setting**	**Criteria**	**Case Definition**	**OCT Device**	**Eye Exam**	**NOS**	**Comments**
Alizadeh[1]	2019	Iran	IP	DSM V	SSD (Acute episode and stable)	Zeiss HD Cirrus 5000 v6.0	Yes	7	No explicit criteria for acute episode vs chronic groups
Altun[2]	2017-2018	Turkey	OP	DSM V	SZ	Optovue RTVue Premier	No	6	Did not specify if acute episodes included
Asanad[3]	2017-2020	USA	OP	DSM V	SZ+SA	Zeiss HD Cirrus 5000 v6.0	No	7	
Bozali[4]	-	Turkey	OP	DSM V	SZ	Nidek RS-3000 Advance v1.5.1	Yes	4	No exclusion of controls with affected 1st degree relatives<br><br>Did not specify if acute episodes included
Celik[5]	-	Turkey	OP	DSM IV	SZ (With and without treatment resistance)	Heidelberg Spectralis v6.0	Yes	5	Pooled SZ subgroups for meta-analysis<br><br>Did not specify if acute episodes included
Delibas[6]	2016-2017	Turkey	OP	DSM IV	SZ (With and without insight)	Zeiss HD Cirrus 4000 v6.5	Yes	6	Pooled SZ subgroups for meta-analysis
Gandu[7]	-	USA	OP	DSM IV	SZ + SA	Heidelberg Spectralis vNR<br><br>Topcon DRI Triton SS OCT vNR	No	6	Heidelberg data used for meta-analysis<br><br>Did not specify if acute episodes included
Hosak[8]	-	Czech Republic	OP	DSM V	SZ	Zeiss HD Cirrus vNR	No	5	Did not specify if acute episodes included
Jerotic[9]	-	Serbia	OP	ICD 10	SZ + SA + Psychosis NOS	Zeiss HD Cirrus 4000 vNR	Yes	5	Pooled gender subgroups for meta-analysis
Kaya[10]	-	Turkey	OP	DSM V	SZ	Optopol REVO vNR	No	7	No exclusion of controls with affected 1st degree relatives<br><br>Did not specify if acute episodes included
Koman-Wierdak[11]	2019-2020	Poland	IP	ICD-10	SZ (In acute remission)	Optovue Avanti vNR	Yes	6	No exclusion of controls with affected 1st degree relatives<br><br>OCT-A study
Kozub[12]	-	Russia	IP + OP	NR	SZ + Schizotypical PD (Majority acute episode)	Topcon 3D OCT 2000	Yes	3	No control description<br><br>83% acute cases, included in acute meta-analysis

Kurt[13]	2019	Turkey	IP+OP	DSM V	SZ + SA	Zeiss HD Cirrus vNR	Yes	5	No exclusion of controls with affected 1st degree relatives<br><br>22.7% Acute cases, not included in acute meta-analysis
Kurtulmus[14]	2017-2018	Turkey	OP	DSM IV	SZ +1st degree unaffected relatives	Heidelberg Spectralis v6.9	No	7	
Lai[15]	-	USA	OP	DSM IV	SZ + SA<br>(Recent first acute episode and stable)	Zeiss HD Cirrus 5000 vNR	No	7	No exclusion of controls with affected 1st degree relatives
Lee[16]	2012-2013	Malaysia	IP + OP	DSM IV	SZ<br>(Mixed acute episode, stable, long-term stable)	Zeiss HD Cirrus 4000 vNR	Yes	8	No exclusion of controls with affected 1st degree relatives<br><br>Partial data reported for acute, chronic and long term chronic.
Liu[17]	2018-2019	China	IP	DSM IV	SZ	Topcon 3D OCT v8.42	Yes	7	No exclusion of controls with affected 1st degree relatives<br><br>Did not specify if acute episodes included
Miller[18]	-	USA	OP	DSM IV	SSD	Zeiss HD Cirrus 4000 v6.5.0.772<br><br>Heidelberg Spectralis v6.0.7	No	3	Cirrus values used for meta-analysis<br><br>Controls recruited from separate study<br><br>Did not specify if acute episodes included
Mota[19]	2014	Portugal	OP	ICD-10	SZ	Heidelberg Spectralis vNR	Yes	5	
Sarkar[20]	2016-2017	India	IP	ICD 10	SZ<br>(Acute episode)	OPKO/OTI Spectral OCT/SLO v4.0	Yes	7	
Schönfeldt-Lecuona[21]	2014-2015	Germany	OP	ICD 10	SZ + SA	Heidelberg Spectralis v6.0	Yes	6	No exclusion of controls with affected 1st degree relatives<br><br>Did not specify if acute episodes included
Silverstein[22]	-	USA	OP	DSM IV	SZ<br>(With and without cardiometabolic disease)	Zeiss HD Cirrus 4000 v8.1.0.117	Yes	6	Pooled SZ subgroups for meta-analysis<br><br>Did not specify if acute episodes included
Topcu-Yilmaz[23]	2014-2015	Turkey	IP	DSM IV	SZ<br>(Acute Episode)	Heidelberg Spectralis vNR	Yes	5	Disease duration reported as proportion
Yilmaz[24]	2014-2015	Turkey	OP	-	SZ	Zeiss HD Cirrus 4000 vNR	Yes	3	No exclusion of controls with affected 1st degree relatives<br><br>Did not specify if acute episodes included

Qualitative Analysis									
**Study**	**Period**	**Country**	**Setting**	**Criteria**	**Case Definition**	**OCT Device**		**NOS**	**Comments**
Bannai[25]	-	USA	IP	DSM IV	SZ + SA	Topcon DRI Triton SS OCT vNR	No	6	OCT-A study
Budakoglu[26]	2018-2019	Turkey	NR	DSM IV	SZ	Optovue RTVue XR Avanti v2017.1.0.151	Yes	5	OCT-A study
Huang[27]	2019	China	IP	DSM IV	SZ (First untreated episode)	Zeiss HD Cirrus 4000 vNR	No	5	Excluded from meta-analysis (Non-compatible OCT data)
Jerotic[28]	-	Serbia	OP	ICD 10	SZ + SA + Psychosis NOS	Zeiss HD Cirrus 4000 vNR	Yes	6	Excluded from meta-analysis (Overlapping cohort)
Liu[29]	2018-2020	China	IP	DSM IV	SZ	Topcon 3D OCT-2000 v8.42	Yes	6	Excluded from meta-analysis (Overlapping cohort)<br><br>No exclusion of controls with affected 1st degree relatives<br><br>Did not specify if acute episodes included
Orum[30]	-	Turkey	OP	DSM V	SZ (With and without treatment resistance) (Clozapine vs first vs second generation antipsychotics)	Heidelberg Spectralis v6.0	Yes	5	Excluded from meta-analysis (Non-compatible OCT data)<br><br>No exclusion of controls with affected 1st degree relatives
Samani[31]	2014-2015	UK	IP + OP	ICD 10	SZ	Leica Envisu hand-held SD-OCT system (Bioptigen)	No	6	Excluded from meta-analysis (Non-compatible OCT data)
Silverstein[32]	-	USA	OP	-	SZ + SA	Zeiss HD Cirrus5000 + AngioPlex Matrix vNR	No	3	OCT-A study
Zhuo[33]	2018-2019	China	IP + OP	DSM IV	SZ (First episode of visual and auditory hallucinations and MRI brain structural impairment)	Zeiss HD Cirrus 4000 vNR	No	6	Pooled SZ subgroups for meta-analysis<br><br>Selective reporting (97 of 113) those with simultaneous brain and retina structural abnormalities
Zhuo[34]	2016-2019	China	IP + OP	DSM IV	SZ (First episode of visual hallucination, with or without auditory hallucination)	Zeiss HD Cirrus 4000 vNR	No	7	Excluded from meta-analysis (Overlapping cohort)
Zhuo[35]	2018-2019	China	IP + OP	DSM IV	SZ (First episode visual and auditory hallucinations)	Zeiss HD Cirrus 4000 vNR	No	7	Excluded from meta-analysis (Duplicate cohort)
Zhuo[36]	2016-2019	China	IP + OP	DSM IV	SZ (First episode of untreated visual hallucinations)	Zeiss HD Cirrus 4000 vNR	No	7	Excluded from meta-analysis (Overlapping cohort, non-compatible OCT data)<br><br>Longitudinal study

Summary of study characteristics meeting selection criteria. Controls were defined as no known personal or first degree relative with psychiatric disease. Eye exam included at least visual acuity, intraocular pressure and anterior/posterior slit lamp microscopy. BPAD: Bipolar affective disorder; DSM: Diagnostic and Statistical Manual of Mental Disorders; ICD: International Classification of Diseases; IP: Inpatient; NOS: Newcastle-Ottawa Scale; NR: Not reported; OCT: Optical coherence tomography; OCT-A: OCT Angiography; OP: Outpatient; SA: Schizoaffective disorder; SSD: Schizophrenia spectrum disorder; SZ: Schizophrenia

Table S4. Summary of Participant Characteristics

Quantitative Analysis										
**Study**	**Group**	**n**	**Age**	**Male**	**PANSS†**	**Duration (years)**	**CPZ mg Equivalent or Treatment‡**	**Smoker**	**Cardio-metabolic disease**	**BMI**
Alizadeh[1]	SSD (Acute Episode)	15	37.7 ±9.2	100%	NR	12.3 ±8.5	NR	NR	0	18 to 28
	SSD (Stable)	15	38.3 ±7.9	100%	NR	15.3 ±9.7	NR	NR	0	18 to 28
	Control	15	36.4 ±9.5	100%	-	-	-	NR	0	18 to 28
Altun[2]	SZ	35	44.2 ±10.0	75%	*SANS 44.5±16.3 SAPS 21.3±14.0*	20.3 ±8.9	283.4 ±141.9	66%*	0	NR
	Control	31	39.4 ±4.3	64%	-	-	-	77%*	0	NR
Asanad[3]	SZ + SA	58	37.2 ±12.3	79%*	NR	NR	(91%)	NR	NR	NR
	Control	35	41.1 ±15.2	46%*	-	-	-	NR	NR	NR
Bozali[4]	SZ	57	37.2 ±9.9	60%	NR	NR	NR	NR	0	NR
	Control	57	36.8 ±9.6	58%	-	-	-	NR	0	NR
Celik[5]	SZ (Resistant)	40	35.8 ±10.7	80%	85.6* ±14.0	14.6 ±8.3	(100%)	NR	0	NR
	SZ (Responsive)	41	35.4 ±9.7	78%	62.4* ±18.3	12.0 ±9.9	(100%)	NR	0	NR
	Control	41	35.5 ±16.0	68%	-	-	-	NR	0	NR
Delibas[6]	SZ (No Insight)	32	39.8 ±11.8	50%	67.3 ±12.4	13.5 ±10.9	694 ±377	NR	0	NR
	SZ (Insight)	31	42.1 ±9.8	58%	52.6 ±12.6	15.8 ±8.8	827 ±485	NR	0	NR
	Control	39	40.1 ±9.0	64%	-	-	-	NR	NR	NR
Gandu[7]	SZ + SA	30	36.2 ±12.7	70%	55.8 ±14.3	12.5 ±12.4	220 ±220	30%*	30%	30.9* ±7.9
	Control	22	37.4 ±11.5	68%	-	-	-	0*	29%	26.3* ±3.9
Hosak[8]	SZ	39	30.5* ±7.1	69%*	NR	9.7±7.1	NR	56%*	0	27.7* ±4.8
	1st degree relative	39	47.2* ±11.5	39%*	-	-	-	28%*	0	26.1* ±4.4
	Control	32	33.4* ±9.1	69%*	-	-	-	19%*	0	24.9* ±4.2
Jerotic[9]	SZ + SA + Psychosis NOS	42	32.6 ±6.9	52%	NR	6.1 ±4.4	211 ±122	NR	0	NR
	Control	39	32.3 ±9.5	49%	-	-	-	NR	0	NR
Kaya[10]	SZ	46	31.5 ±8.1	70%	69.2 ±15.2	12.0 ±6.9	(100%)	Yes	NR	26.9 ±4.9
	1st degree sibling	46	30.4 ±9.0	57%	-	-	-	Yes	NR	24.7 ±4.5
	Control	46	32.0 ±8.0	70%	-	-	-	Yes	NR	26.3 ±4.5
Koman-Wierdak[11]	SZ (Acute remission)	12	26.3 ±5.3	50%	NR	3.5 ±3.5	720 ±NR	NR	0	23 ±NR
	Control	15	26.8 ±3.4	47%	-	-	-	NR	0	24 ±NR

Kozub[12]	SZ + Schizo-typical PD<br>(Majority Acute Episode)	12	27.6<br>(18-37)	67%	NR	8.5<br>±5.4	(100%)	NR	NR	NR
	Control	12	28.3<br>(19-36)	50%	-	-	-	NR	NR	NR
Kurt[13]	SA+SZ<br>(Mixed Acute, Stable)	44	47.8<br>±9.4	68%	102.3<br>±16.6	24.8<br>±15.2	(100%)	NR	NR	NR
	Control	41	45.6<br>±9.9	68%	-	-	-	NR	NR	NR
Kurtulmus[14]	SZ	38	41.0<br>±11.3	53%	66.4<br>±14.6	18.3<br>±9.7	781<br>±478	42%	0	26.6<br>±5.0
	1st degree relative	38	42.1<br>±13.9	29%	-	-	-	29%	0	27.3<br>±6.5
	Control	38	38.5<br>±11.2	53%	-	-	-	53%	0	26.4<br>±4.5
Lai[15]	SZ+SA<br>(Recent First Episode)	15	24.6<br>±6.5	93%*	NR	NR	214<br>±239	NR	0	NR
	Control<br>(<30yo)	20	23.1<br>±2.3	45%*	-	-	-	NR	0	NR
	SZ+SA<br>(Stable)	18	39.7<br>±11.0	78%	NR	NR	213<br>±280	NR	0	NR
	Control<br>(>30yo)	18	41.9<br>±11.8	83%	-	-	-	NR	0	NR
Lee[16]	SZ<br>(Mixed Acute Episode, Stable, Long-term Stable)	30	37.2<br>±10.7	60%	NR	*16.7% <2yrs*<br>*43.3% 2-10yrs*<br>*40.0% >10yrs*	NR	NR	0	NR
	Control	30	36.0<br>±9.1	47%	-	-	-	NR	0	NR
Liu[17]	SZ	221	44.2*<br>±12.0	56%*	NR	20.1<br>±12.5	52<br>±13	24%*	0	24.2<br>±4.4
	Control	149	41.6*<br>±9.4	64%*	-	-	-	9%*	0	23.9<br>±3.3
Miller[18]	SSD	12	48.3<br>±10.3	67%	NR	27.3<br>±14.4	551<br>±454	NR	NR	NR
	Control	12	48.3<br>±10.6	67%	-	-	-	NR	NR	NR
Mota[19]	SZ	20	32.9<br>±11.9	85%	NR	NR	NR	NR	0	NR
	Control	20	33.4<br>±11.2	80%	-	-	-	NR	0	NR
Sarkar[20]	SZ<br>(Acute Episode)	20	28.6<br>±6.3	70%	73.4<br>±8.1	2.5<br>±0.9	NR	NR	0	NR
	Control	20	30.3<br>±9.2	60%	-	-	-	NR	0	NR
Schönfeldt-Lecuona[21]	SZ + SA	26	37.0<br>±10.9	65%	*PANSS-P 14.9±4.4*<br>*PANSS-N 18.8±4.4*	10.2<br>±10.2	(100%)	NR	0	27.9<br>±5.0
	Control	23	40.3<br>±11.6	52%	-	-	-	NR	0	25.6<br>±3.7
Silverstein[22]	SZ	32	40.5<br>±12.1	59%	NR	NR	462<br>±NR	NR	34%	NR
	Control	32	39.2<br>±11.0	56%	-	-	-	NR	34%	NR
Topcu-Yilmaz[23]	SZ<br>(Acute Episode)	59	34.6<br>±9.5	54%	75.2<br>±20.1	10.3<br>±NR<br>*13.6% 'acute'*<br>*86.4% 'chronic'*	(81%)	NR	0	NR
	Control	37	32.1<br>±12.3	41%	-	-	-	NR	0	NR

Yilmaz[24]	SZ	34	39.9 ±10.3	56%	NR	NR	NR	0	0	NR
	Control	30	38.6 ±9.6	44%	-	-	-	0	0	NR
Qualitative Analysis										
**Study**	**Group**	**n**	**Age**	**Male**	**PANSS**	**Duration (years)**	**CPZ mg Equivalent or Treatment**	**Smoker**	**Cardio-metabolic disease**	**BMI**
Bannai[25]	SZ + SA	26	37 ±12.7	77%	55.5 ±14.6	13.6 ±12.8	339 ±256	31%*	27%	30.5 ±8.0
	Control	21	38 ±11.6	67%				0%*	30%	26.3 ±4.0
Budakoglu[26]	SZ	22	40.8 ±7.9	64%	NR	8.3 ±3.3	NR	NR	0	NR
	Control	26	45.2 ±8.1	69%	-	-	-	NR	0	NR
Huang[27]	SZ (First untreated episode)	100	23.0 ±2.5	56%	80.9 ±12.5	0.2 ±0.06	NR	NR	NR	NR
	Control	100	23.5 ±3.0	56%	-	-	-	NR	NR	NR
Jerotic[28]	SZ + SA + Psychosis NOS	33	33.1 ±6.1	53%	13.3 ±3.1	5.9 ±3.9	220 ±132	NR	0	NR
	Control	35	32.5 ±9.9	51%	-	-	-	NR	0	NR
Liu[29]	SZ	138	45.09* ±12.47	52%*	80.4 ±31.8	21.7 ±13.5	(100%)	27%*	0	24.3 ±4.2
	Control	160	41.43* ±9.5	34%*	-	-	-	6%*	0	23.4 ±3.2
Orum[30]	SZ (Clozapine)	26	33.0 ±8.3	78%	78.9 ±16.0	12.7 ±8.0	(100%)	NR	0	NR
	SZ (1st Gen Antipsychotic)	22	41.4 ±10.7		75.6 ±11.6	18.0 ±9.8	(100%)	NR	0	NR
	SZ (2nd Gen Antipsychotic)	34	36.7 ±10.9		69.9 ±18.5	13.6 ±10.2	(100%)	NR	0	NR
	Control	50	41.0 ±13.7	64%	-	-	-	NR	0	NR
Samani[31]	SZ	35	40.6 ±12.9	71%	56.3 ±12.3	16.3 ±9.1	388 ±273	NR	NR	NR
	Control	50	40.6 ±12.7	70%	-	-	-	NR	NR	NR
Silverstein[32]	SZ + SA	28	32.2 ±11.3	86%	NR	NR	236 ±266	NR	0	NR
	Control	37	32.2 ±12.6	65%	-	-	-	NR	0	NR
Zhuo[33]	SZ (Severe AH +Severe VH)	20	22.0* ±4.2	55%*	78.9* ±1.5	0.2* ±0.2	0	NR	0	NR
	SZ (Mod AH +Severe VH)	23	26.4* ±3.0	52%*	80.1* ±6.8	0.3* ±0.2	0	NR	0	NR
	SZ (Severe AH + Mod VH)	28	25.2* ±1.2	50%*	79.5* ±5.9	0.4* ±0.1	0	NR	0	NR
	SZ (Mod AH +Mod VH)	26	27.9* ±3.9	58%*	78.6* ±9.9	0.5* ±0.2	0	NR	0	NR
	Control	30	25.4* ±0.5	50%*	-	-	-	NR	0	NR
Zhuo[34]	SZ (VH + AH)	30	23.7 ±2.5	47%	79.2 ±4.5	NR	0	NR	0	NR
	SZ (VH + No AH)	30	24.5 ±3.1	43%	78.4 ±3.6	NR	0	NR	0	NR

	Control	30	24.0 ±3.8	50%	-	-	-	NR	0	NR
Zhuo[35]	SZ (Severe AH +Severe VH)	20	22.0* ±4.2	55%*	78.9* ±1.5	0.2* ±0.2	0	NR	0	NR
	SZ (Mod AH +Severe VH)	23	26.4* ±3.0	52%*	80.1* ±6.8	0.3* ±0.2	0	NR	0	NR
	SZ (Severe AH + Mod VH)	28	25.2* ±1.2	50%*	79.5* ±5.9	0.4* ±0.1	0	NR	0	NR
	SZ (Mod AH +Mod VH)	26	27.9* ±3.9	58%*	78.6* ±9.9	0.5* ±0.2	0	NR	0	NR
	Control	30	25.4* ±0.5	50%*	-	-	-	NR	0	NR
Zhuo[36]	SZ (VH)	48	21.5 1.7	48%	80.9 12.5	0	0	NR	0	NR
	Control	50	22.0 1.7	50%	-	-	-	NR	0	NR

Summary of participant characteristics of included studies. Numbers presented as mean ± standard deviation (percentage). *Statistically significant difference; †: Symptom severity score based on Positive and Negative Syndrome Scale (PANSS) unless reported otherwise. ‡: Medication reported as mean chlorpromazine (CPZ) mg equivalent, or if not reported, then a percentage receiving treatment is recorded in parenthesis; AH: Auditory hallucinations; BPAD: Bipolar affective disorder; CPZ: Chlorpromazine; NOS: Not otherwise specified; NR: Not reported; SA: Schizoaffective disorder; SANS: Scale for the Assessment of Negative Symptoms; SAPS: Scale for the Assessment of Positive Symptoms; SSD: Schizophrenia spectrum disorder; SZ: Schizophrenia; VH: Visual hallucinations.

Global Peripapillary Retinal Nerve Fibre Layer Additional Figures

Study	SSD Total	SSD Mean	SSD SD	Control Total	Control Mean	Control SD	MD	95%−CI	Weight
Liu 2020	221	88.2	25.8	149	102.1	14.3	−13.95	[−18.06; −9.84]	0.0%
Lee 2013	30	94.7	9.9	30	103.5	6.5	−8.83	[−13.07; −4.59]	6.1%
Alizadeh 2021	30	85.0	12.3	15	91.1	8.9	−6.02	[−12.34; 0.30]	3.5%
Yilmaz 2015	34	87.7	7.3	30	93.2	7.6	−5.53	[−9.18; −1.88]	7.2%
Asanad 2021	58	91.8	9.3	35	96.6	10.4	−4.80	[−9.00; −0.60]	6.2%
Celik 2016	81	101.5	9.4	41	106.3	7.8	−4.77	[−7.93; −1.61]	8.3%
Kurt 2022	44	87.0	9.6	41	91.8	8.1	−4.74	[−8.52; −0.96]	6.9%
Hosak 2020	39	92.7	10.0	32	96.8	6.9	−4.03	[−7.98; −0.08]	6.6%
Altun 2020	35	97.0	13.2	31	100.8	15.5	−3.81	[−10.81; 3.19]	3.0%
Sarkar 2021	20	102.1	11.9	20	105.1	7.3	−3.03	[−9.16; 3.10]	3.7%
Silverstein 2017	32	87.4	20.0	32	89.4	11.7	−2.01	[−10.02; 6.00]	2.4%
Schonfelt−Lecuona 2020	24	97.0	10.0	23	99.0	11.0	−2.00	[−8.02; 4.02]	3.8%
Kurtulumus 2020	38	98.8	9.5	38	100.7	8.7	−1.98	[−6.08; 2.12]	6.3%
Kaya 2022	46	121.6	13.2	46	122.4	13.1	−0.81	[−6.18; 4.56]	4.4%
Miller 2020	10	90.9	11.0	12	91.3	7.8	−0.43	[−8.52; 7.66]	2.3%
Mota 2015	20	101.0	7.3	20	101.2	9.1	−0.15	[−5.27; 4.97]	4.7%
Topcu−Yilmaz 2019	59	101.3	8.5	37	101.3	7.0	0.05	[−3.08; 3.18]	8.4%
Jerotic 2021	42	93.6	8.9	39	93.5	8.1	0.12	[−3.58; 3.82]	7.1%
Lai 2020	33	95.5	9.4	38	94.7	14.2	0.79	[−4.76; 6.34]	4.2%
Kozub 2020	12	96.6	6.7	12	93.9	7.8	2.75	[−3.08; 8.58]	3.9%
Koman−Wierdak 2021	12	118.1	23.6	15	112.6	9.0	5.52	[−8.57; 19.61]	0.9%
**Random effects model**	**920**			**736**			**−2.76**	**[−4.15; −1.36]**	**100.0%**
**Prediction interval**								**[−6.66; 1.14]**	

Heterogeneity: I^2 = 34%, τ^2 = 2.9782, p = 0.07

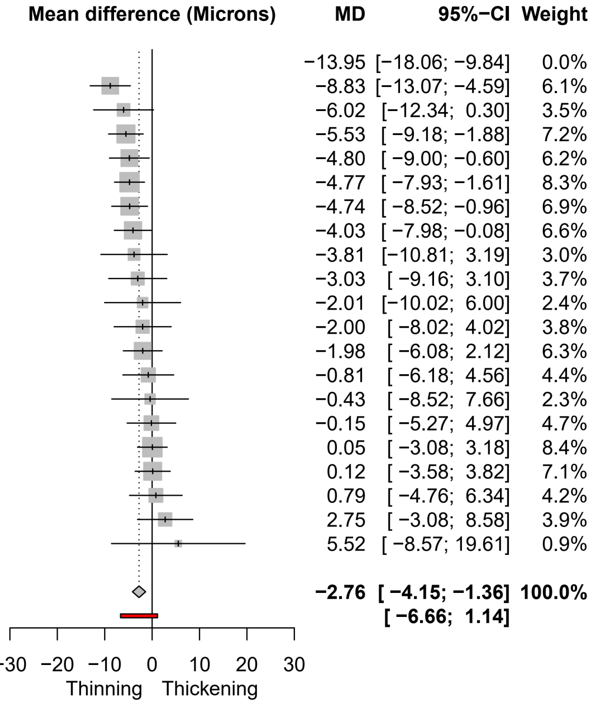

Figure S1. Pooled global pRNFL thickness with removal of outliers detected on influence and GOSH plot analysis

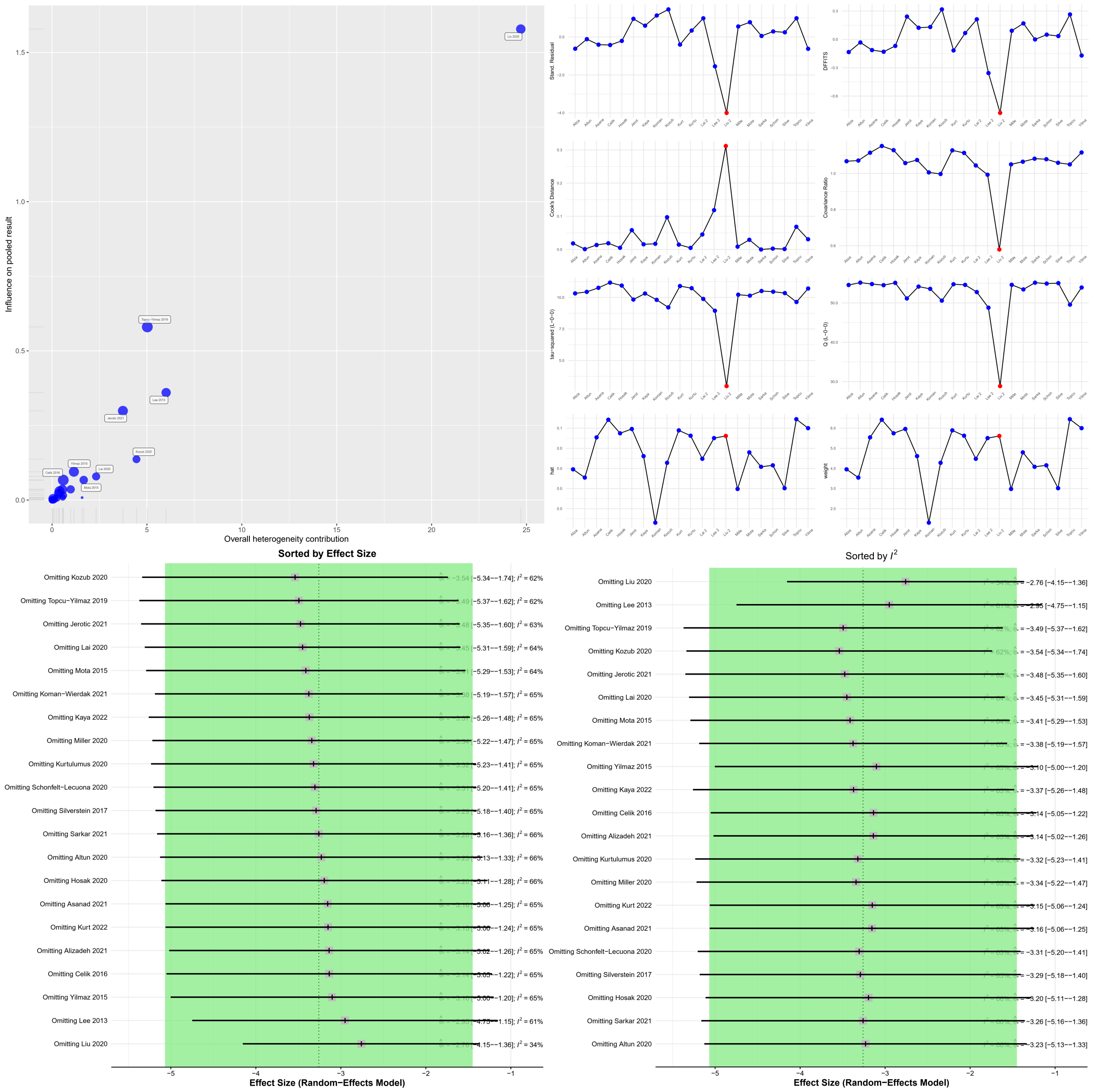

Figure S2. Global pRNFL study influence and outlier analysis. Top left: Baujat plot; Top right: Influence plots; Bottom left: Leave one out analysis sorted by effect size; Bottom right: Leave one out analysis sorted by I^2

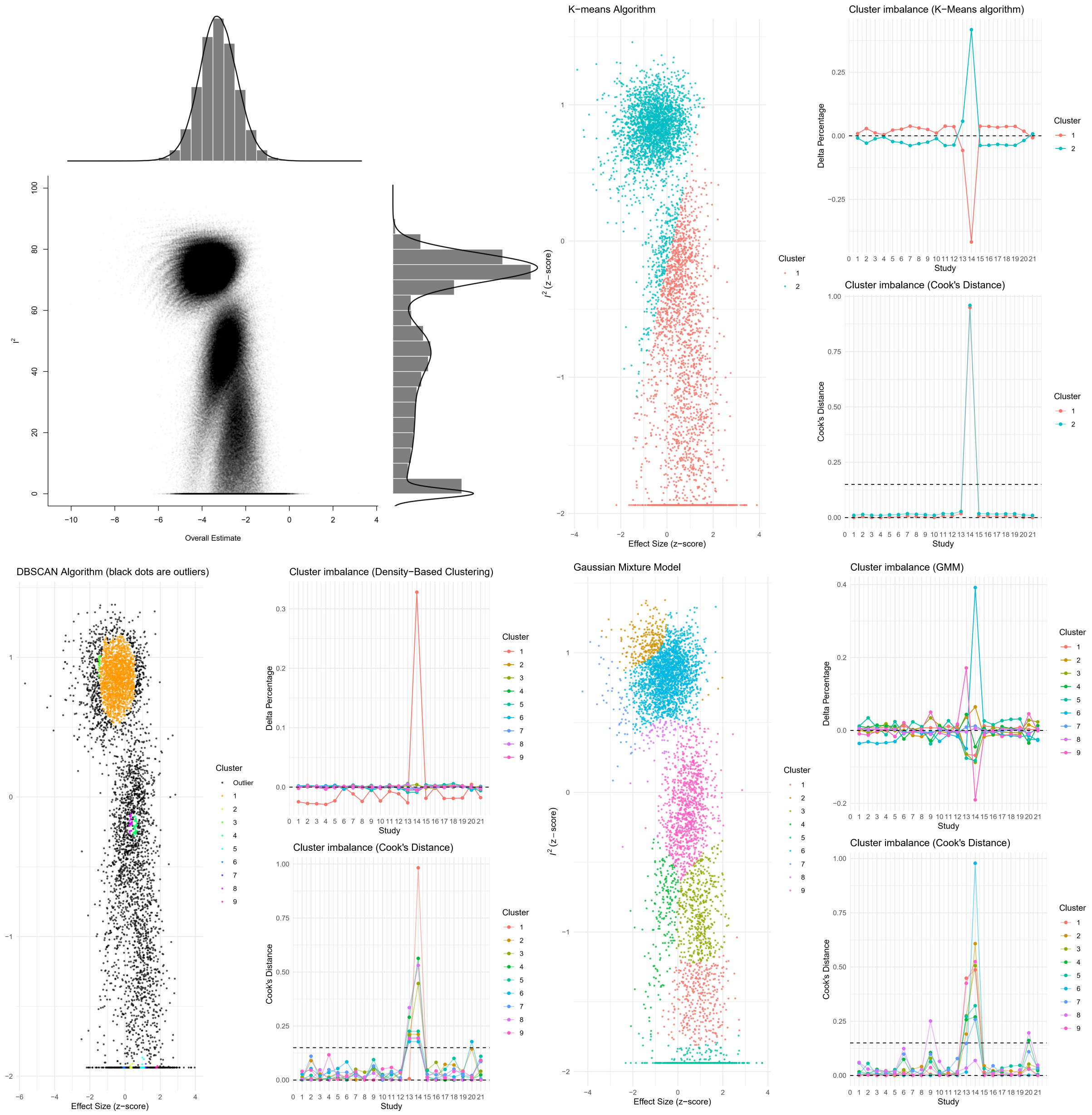

Figure S3. Global pRNFL study GOSH plot heterogeneity analysis. Top left: Raw GOSH plot; Top right: K-means algorithm; Bottom left: DBSCAN algorithm; Bottom right: Gaussian Mixture Model

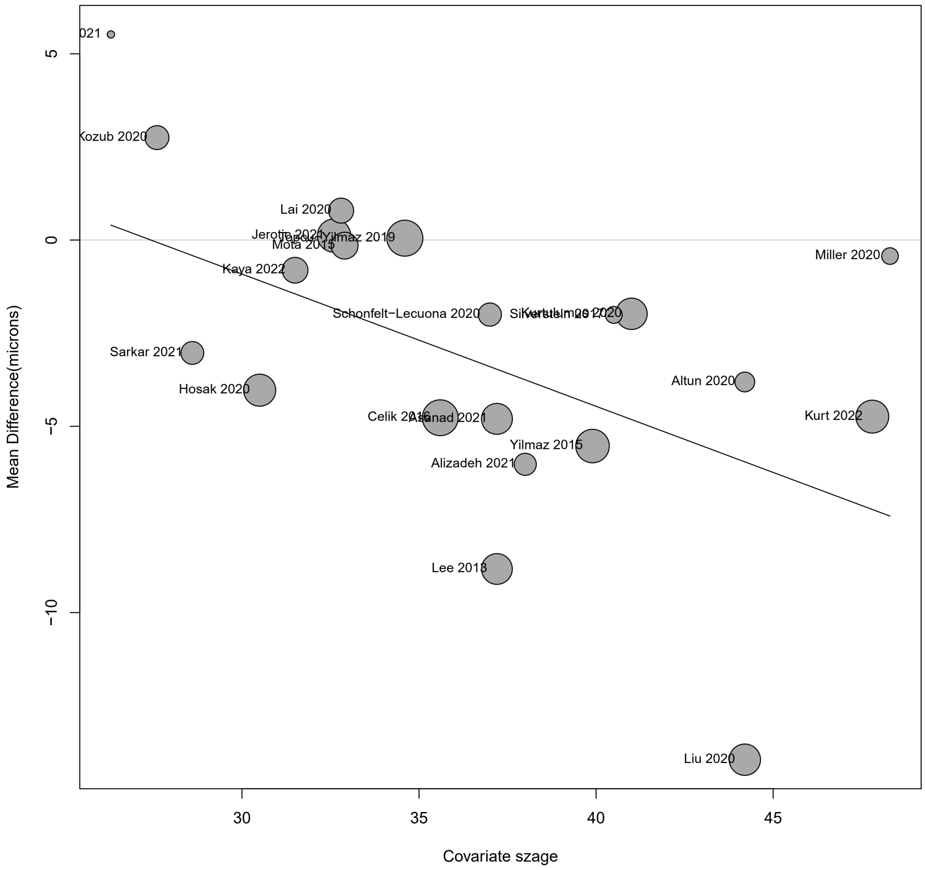

Figure S4. Metaregression mixed effects modelling for global peripapillary retinal nerve fibre layer thinning (microns) in schizophrenia spectrum disorder (SSD) based on average age of SDD cases

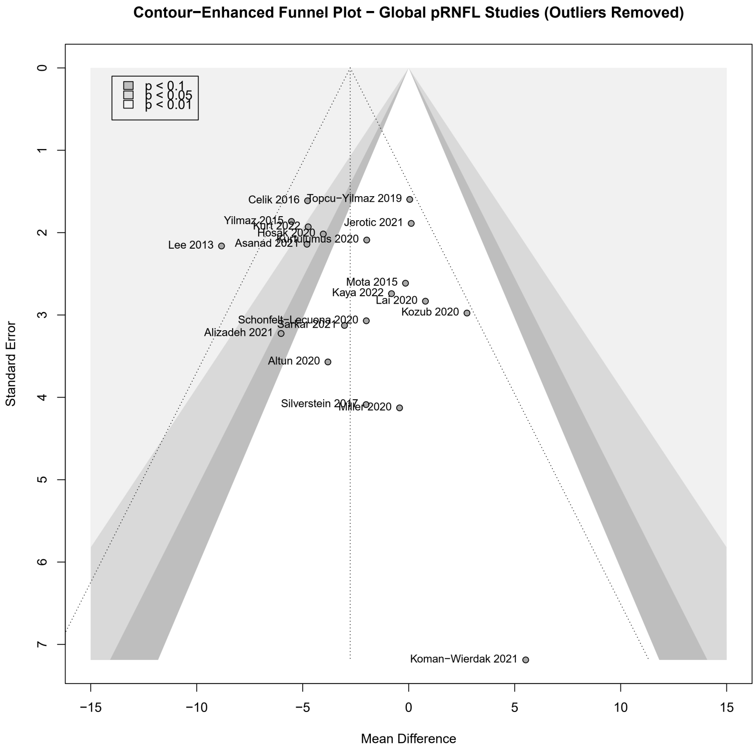

Figure S5. Contour enhanced funnel plot for global peripapillary retinal nerve fibre layer thickness (pRNFL) studies with outliers removed

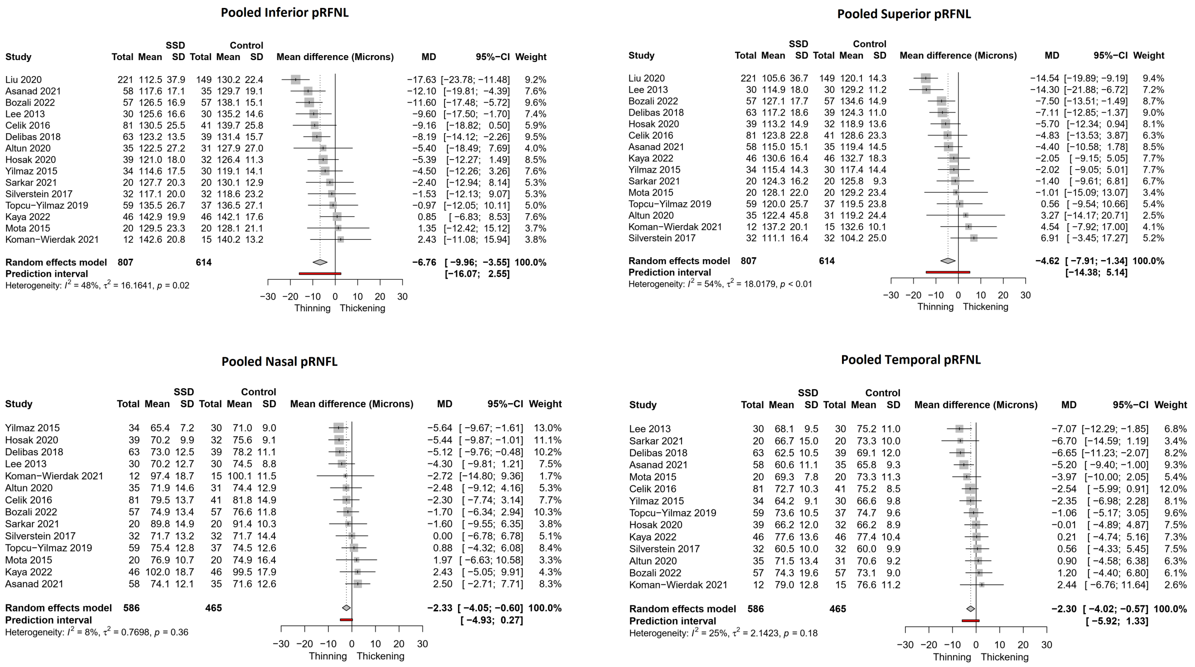

Figure S6. Forest plots of sectoral peripapillary retinal nerve fibre layer (pRNFL) differences (microns) between schizophrenia spectrum disease (SSD) cases and controls

Macular Thickness Additional Figures

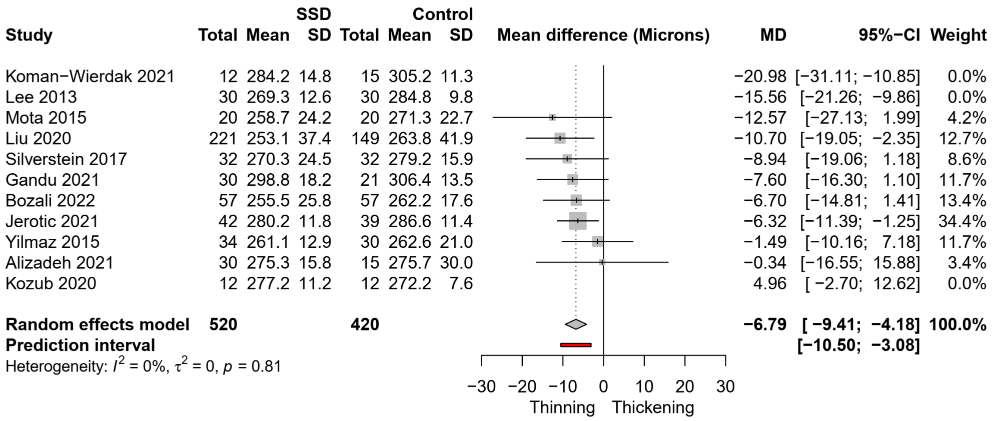

Figure S7. Forest plot of average macular thickness with removal of outliers detected on influence and GOSH plot analysis

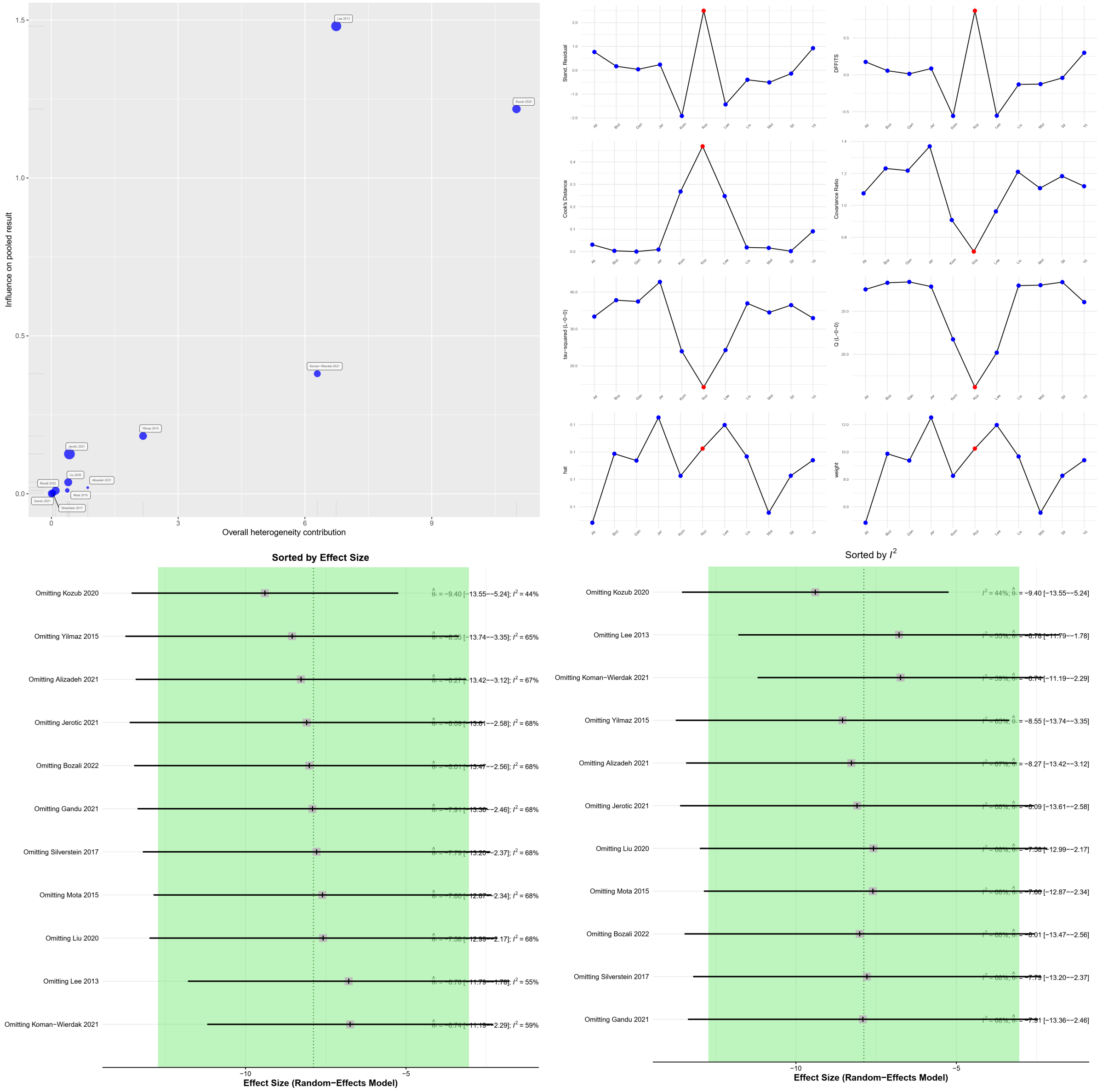

Figure S8. Average macular thickness influence and outlier analysis. Top left: Baujat plot; Top right: Influence plots; Bottom left: Leave one out analysis sorted by effect size; Bottom right: Leave one out analysis sorted by I^2

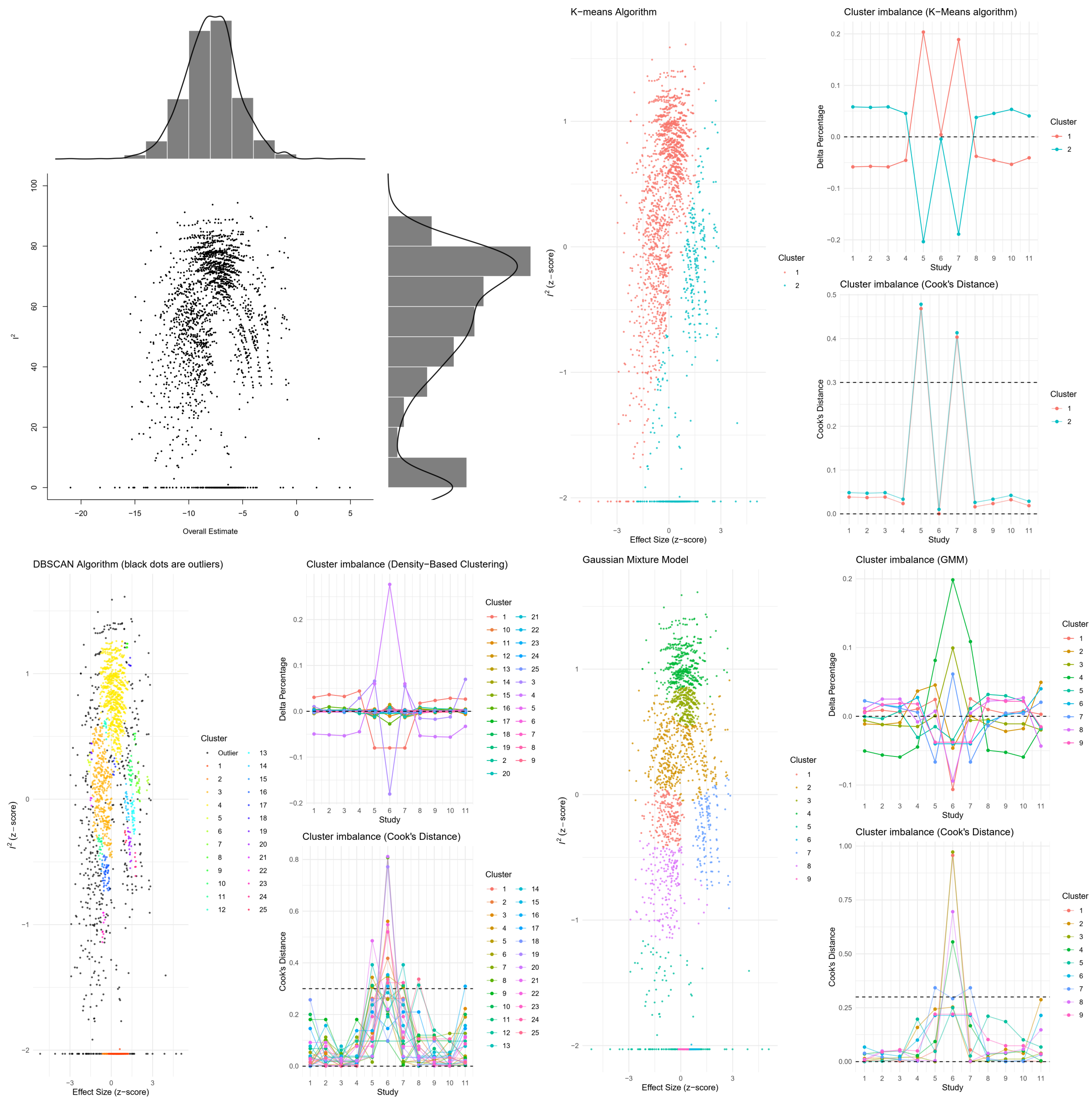

Figure S9. Average macular thickness GOSH plot heterogeneity analysis. Top left: Raw GOSH plot; Top right: K-means algorithm; Bottom left: DBSCAN algorithm; Bottom right: Gaussian Mixture Model

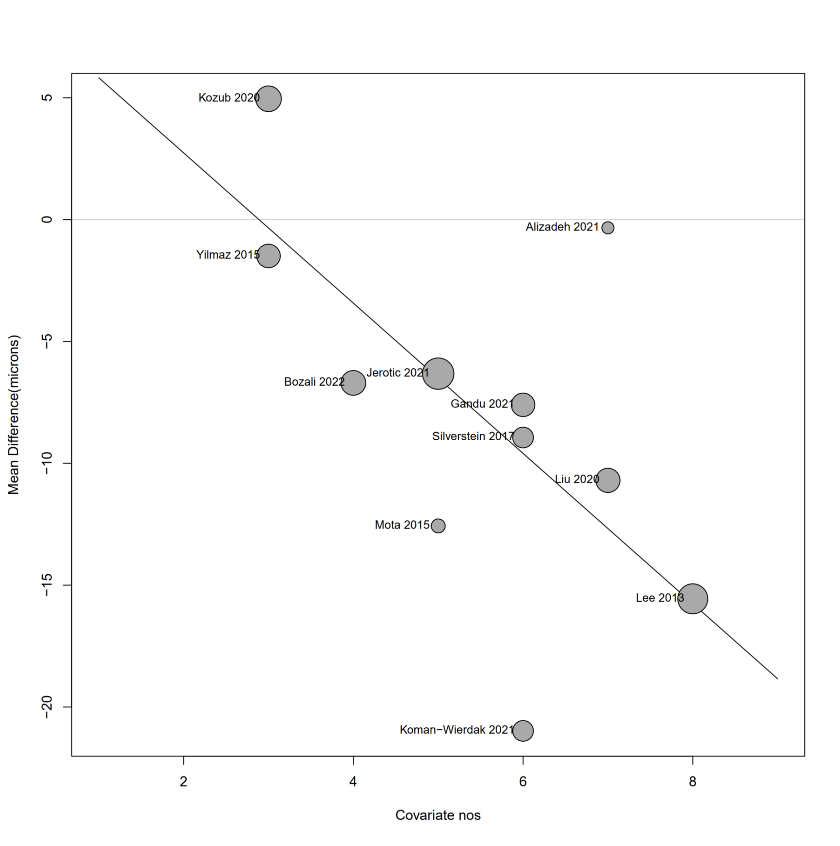

Figure S10. Metaregression mixed effects modelling for average macular thickness mean thinning (microns) in schizophrenia spectrum disorder compared to controls based on the Newcastle-Ottawa Scale of study quality.

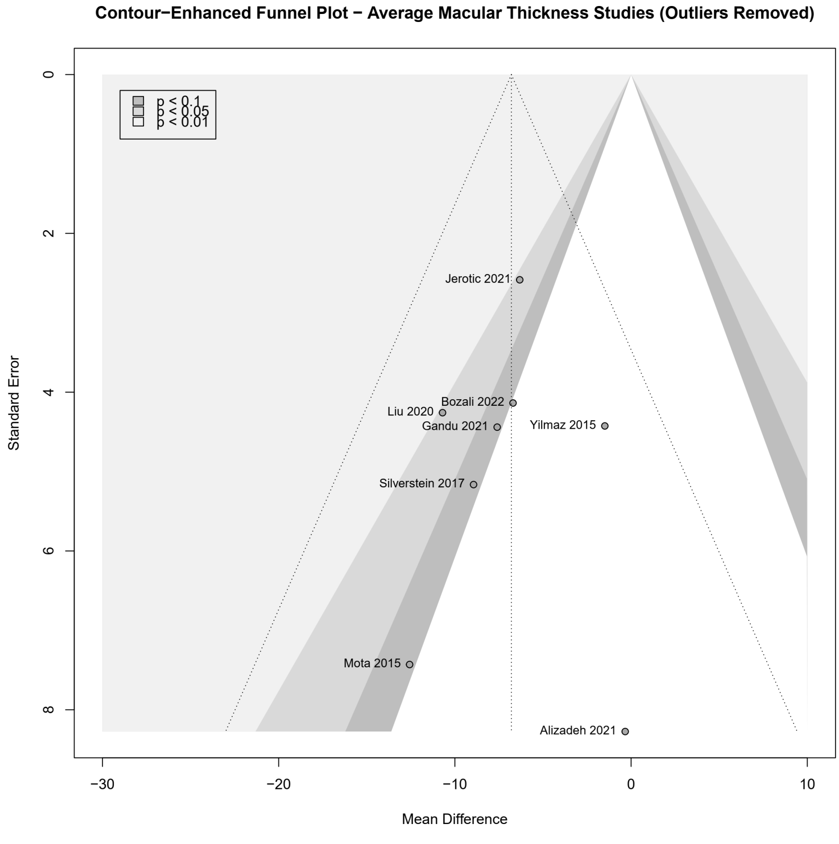

Figure S11. Contour enhanced funnel plot for average macular thickness studies with outliers removed

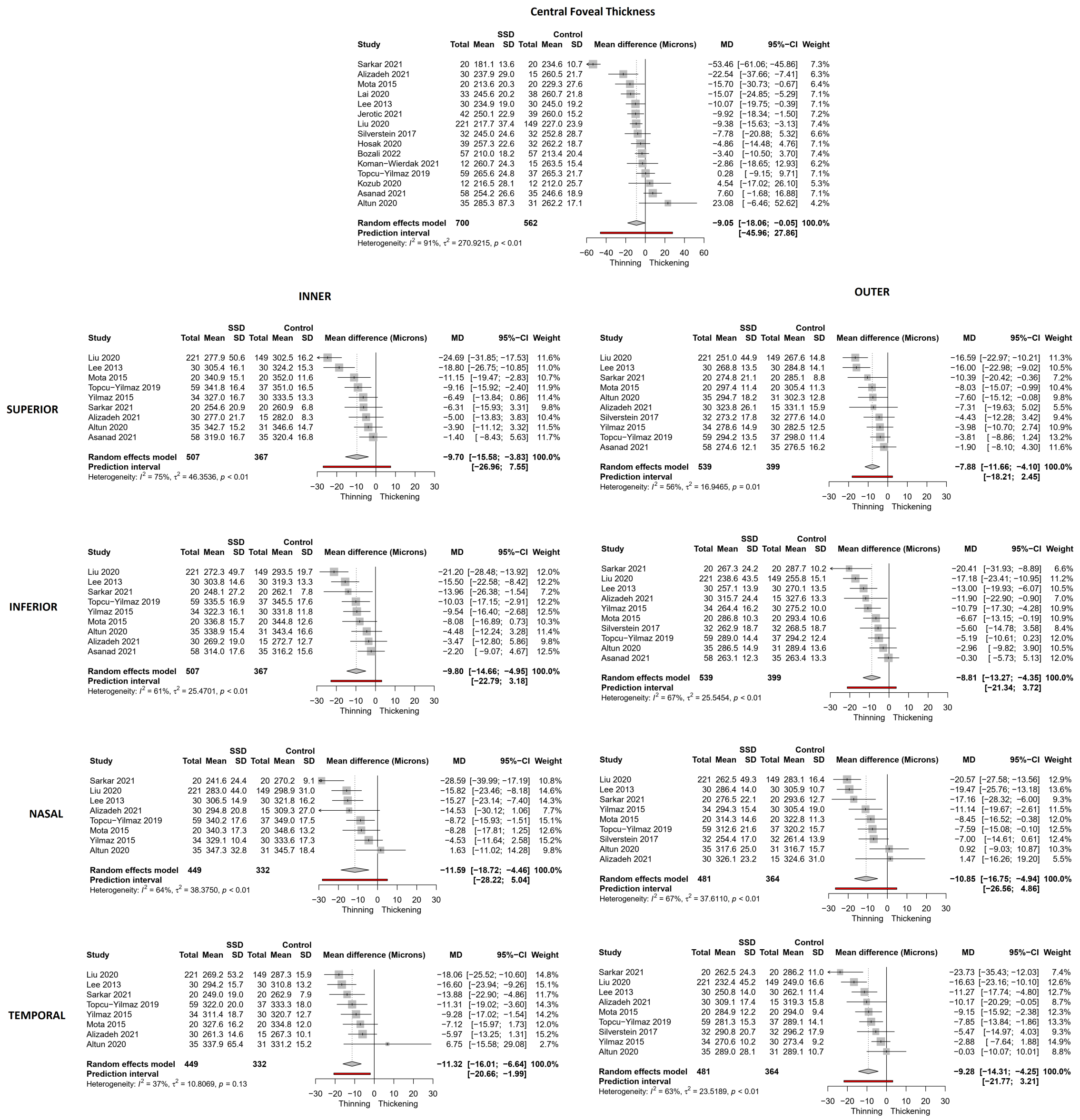

Figure S12. Forest plots of macular thickness differences (microns) between schizophrenia spectrum disease (SSD) cases and controls according to the Early Treatment of Diabetic Retinopathy Study (ETDRS) subfields

Optical Coherence Tomography Readouts

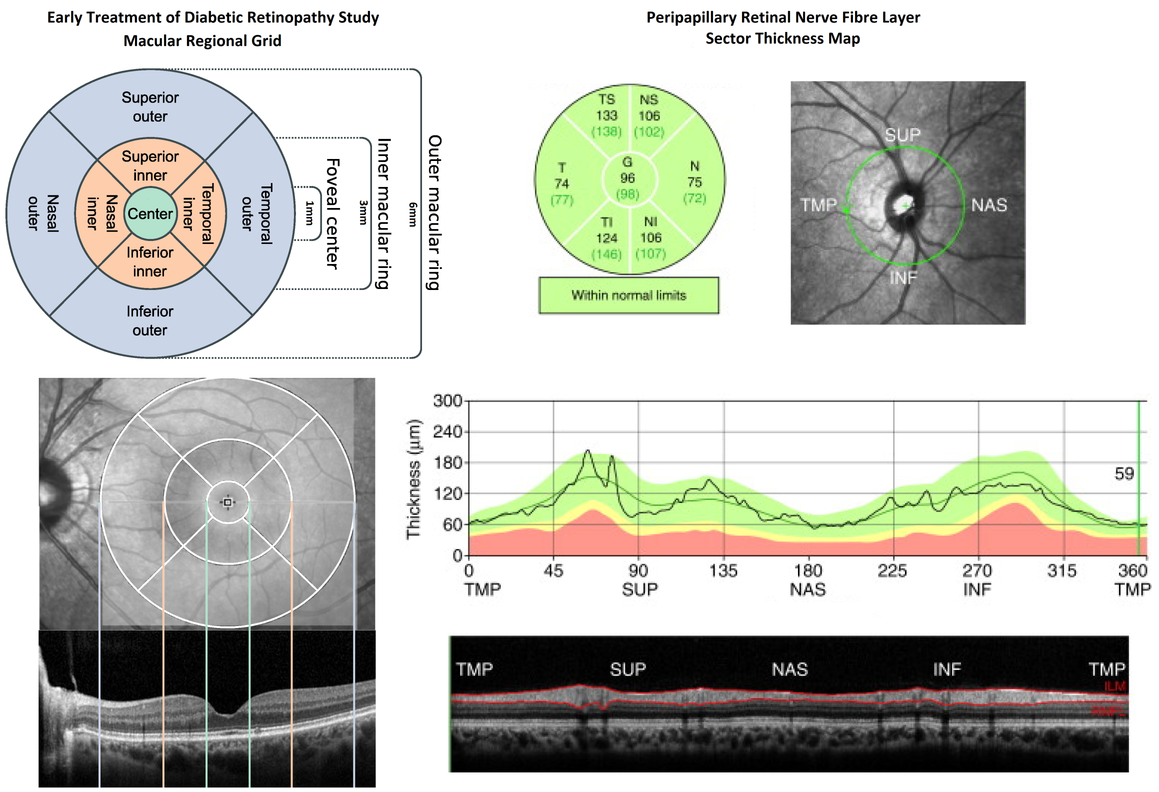

Figure S13. Left: Schematic of the Early Treatment of Diabetic Retinopathy Study (ETDRS) standardised grid for macular thickness measurements. The ETDRS ring diameters are 1, 3 and 6mm respectively with the average total macular thickness reported in each subfield. Right: Schematic of the peripapillary nerve fibre layer (pRNFL) OCT scan. pRNFL scan diameters range between 3.46mm (Cirrus) and 3.45mm (Spectralis).[37] The average thickness can be reported in a schematic grid in each sector from the optic disc, additional subdivisions can be made for each quadrant (such as dividing the superior quadrant to temporal superior (TS) and nasal superior (NS))

Supplementary References

1. Alizadeh M, Delborde Y, Ahmadpanah M, et al. Non-linear associations between retinal nerve fibre layer (RNFL) and positive and negative symptoms among men with acute and chronic schizophrenia spectrum disorder. *J Psychiatr Res*. Sep 2021;141:81-91. doi:10.1016/j.jpsychires.2021.06.007
2. Altun IK, Turedi N, Aras N, Atagun MI. Psychopharmacological Signatures in the Retina in Schizophrenia and Bipolar Disorder: An Optic Coherence Tomography Study. *Psychiatr Danub*. Autumn-Winter 2020;32(3-4):351-358. doi:10.24869/psyd.2020.351
3. Asanad S, O'Neill H, Addis H, et al. Neuroretinal Biomarkers for Schizophrenia Spectrum Disorders. *Transl Vis Sci Technol*. Apr 1 2021;10(4):29. doi:10.1167/tvst.10.4.29
4. Bozali E, Yalinbas D. Analysis of the Thickness of the Outer Retinal Layer Using Optical Coherence Tomography - A Predictor of Visual Acuity in Schizophrenia. Article. *Klinische Monatsblatter fur Augenheilkunde*. 2022;doi:10.1055/a-1741-7988
5. Celik M, Kalenderoglu A, Sevgi Karadag A, Bekir Egilmez O, Han-Almis B, Şimşek A. Decreases in ganglion cell layer and inner plexiform layer volumes correlate better with disease severity in schizophrenia patients than retinal nerve fiber layer thickness: Findings from spectral optic coherence tomography. *Eur Psychiatry*. Feb 2016;32:9-15. doi:10.1016/j.eurpsy.2015.10.006
6. Delibas DH, Karti O, Erdogan E, Sahin T, Bilgic O, Erol A. Decreases in retinal nerve fiber layer and ganglion cell-inner plexiform layer thickness in schizophrenia, relation to insight: A controlled study. [Turkish]. Sizofrenide retina sinir lifi ve ganglion hucre-ic pleksiform tabaka kalinliklarindaki azalma, ic goru ile iliskisi: Kontrollu bir calisma. *Anadolu Psikiyatr Derg*. 2018;19(3):264-273. doi:http://dx.doi.org/10.5455/apd.276720
7. Gandu S, Bannai D, Adhan I, et al. Inter-device reliability of swept source and spectral domain optical coherence tomography and retinal layer differences in schizophrenia. *Biomarkers in Neuropsychiatry*. December 2021;5 (no pagination)100036. doi:http://dx.doi.org/10.1016/j.bionps.2021.100036
8. Hosak L, Zeman T, Studnicka J, et al. Retinal arteriolar and venular diameters are widened in patients with schizophrenia. *Psychiatry Clin Neurosci*. Nov 2020;74(11):619-621. doi:10.1111/pcn.13123
9. Jerotic S, Lalovic N, Pejovic S, et al. Sex differences in macular thickness of the retina in patients with psychosis spectrum disorders. SZ Original - Unable to retrieve. *Prog Neuropsychopharmacol Biol Psychiatry*. Aug 30 2021;110:110280. doi:10.1016/j.pnpbp.2021.110280
10. Kaya H, Ayık B, Tasdelen R, Sevimli N, Ertekin E. Comparing retinal changes measured by optical coherence tomography in patients with schizophrenia and their siblings with healthy controls: Are retinal findings potential endophenotype candidates? Article. *Asian J Psychiatry*. 2022;72103089. doi:10.1016/j.ajp.2022.103089
11. Koman-Wierdak E, Róg J, Brzozowska A, et al. Analysis of the peripapillary and macular regions using OCT angiography in patients with schizophrenia and bipolar disorder. Article. *Journal of Clinical Medicine*. 2021;10(18)4131. doi:10.3390/jcm10184131
12. Kozub KE, Shelepin IE, Chomskii AN, Sharybin EA, Ivanova EA. A structural and functional study of the retina in patients with schizophrenia. Article. *Oftalmol Zh*. 2020;(4):38-44. doi:10.31288/oftalmolzh202043844
13. Kurt A, Zor KR, Küçük E, Yıldırım G, Erşan EE. An Optical Coherence Tomography Study that Supports the Neurovascular Basis of Schizophrenia Disease. Article. *Anadolu Psikiyatr Derg*. 2022;23(1):12-17. doi:10.5152/alphapsychiatry.2021.21207
14. Kurtulmus A, Elbay A, Parlakkaya FB, Kilicarslan T, Ozdemir MH, Kirpinar I. An investigation of retinal layer thicknesses in unaffected first-degree relatives of schizophrenia patients. *Schizophr Res*. Apr 2020;218:255-261. doi:10.1016/j.schres.2019.12.034
15. Lai A, Crosta C, Loftin M, Silverstein SM. Retinal structural alterations in chronic versus first episode schizophrenia spectrum disorders. *Biomarkers in Neuropsychiatry*. June 2020;2 (no pagination)100013. doi:http://dx.doi.org/10.1016/j.bionps.2020.100013
16. Lee WW, Tajunisah I, Sharmilla K, Peyman M, Subrayan V. Retinal nerve fiber layer structure abnormalities in schizophrenia and its relationship to disease state: evidence from optical

coherence tomography. *Invest Ophthalmol Vis Sci*. Nov 21 2013;54(12):7785-92. doi:10.1167/iovs.13-12534
17. Liu Y, Huang L, Tong Y, Chen J, Gao D, Yang F. Association of retinal nerve fiber abnormalities with serum CNTF and cognitive functions in schizophrenia patients. *PeerJ*. 2020;8:e9279. doi:10.7717/peerj.9279
18. Miller M, Zemon V, Nolan-Kenney R, et al. Optical coherence tomography of the retina in schizophrenia: Inter-device agreement and relations with perceptual function. *Schizophr Res*. May 2020;219:13-18. doi:10.1016/j.schres.2019.10.046
19. Mota M PP, Klut C, Coutinho I, Santos C, Pires G, Maia T, Melo A. Evaluation of Structural Changes in the Retina of Patients with Schizophrenia. *Ophthalmology Research: An International Journal*. 2015;4(2):45-52.
20. Sarkar S, Rajalakshmi AR, Avudaiappan S, Eswaran S. Exploring the role of macular thickness as a potential early biomarker of neurodegeneration in acute schizophrenia. *Int Ophthalmol*. Aug 2021;41(8):2737-2746. doi:10.1007/s10792-021-01831-z
21. Schönfeldt-Lecuona C, Kregel T, Schmidt A, et al. Retinal single-layer analysis with optical coherence tomography (OCT) in schizophrenia spectrum disorder. *Schizophr Res*. May 2020;219:5-12. doi:10.1016/j.schres.2019.03.022
22. Silverstein SM, Paterno D, Cherneski L, Green S. Optical coherence tomography indices of structural retinal pathology in schizophrenia. *Psychol Med*. Sep 2018;48(12):2023-2033. doi:10.1017/s0033291717003555
23. Topcu-Yilmaz P, Aydin M, Cetin Ilhan B. Evaluation of retinal nerve fiber layer, macular, and choroidal thickness in schizophrenia: spectral optic coherence tomography findings. *Psychiatry and Clinical Psychopharmacology*. 02 Jan 2019;29(1):28-33. doi:http://dx.doi.org/10.1080/24750573.2018.1426693
24. Yılmaz U, Küçük E, Ülgen A, et al. Retinal nerve fiber layer and macular thickness measurement in patients with schizophrenia. *Eur J Ophthalmol*. Jun 10 2016;26(4):375-8. doi:10.5301/ejo.5000723
25. Bannai D, Adhan I, Katz R, et al. Quantifying Retinal Microvascular Morphology in Schizophrenia Using Swept-Source Optical Coherence Tomography Angiography. Article. *Schizophr Bull*. 2022;48(1):80-89. doi:10.1093/schbul/sbab111
26. Budakoglu O, Ozdemir K, Safak Y, Sen E, Taskale B. Retinal nerve fibre layer and peripapillary vascular density by optical coherence tomography angiography in schizophrenia. *Clin Exp Optom*. Feb 25 2021:1-7. doi:10.1080/08164622.2021.1878816
27. Huang J, Song X, Xu Y, et al. Reliability and Diagnostic Validity of A Novel Visual Disturbance Subjective Experience Scale in Chinese Patients with Schizophrenia. *Psychiatry and Clinical Psychopharmacology*. August 2020;30(3):307-312. doi:https://dx.doi.org/10.5455/PCP.20200302022126
28. Jerotic S, Ristic I, Pejovic S, et al. Retinal structural abnormalities in young adults with psychosis spectrum disorders. *Prog Neuropsychopharmacol Biol Psychiatry*. Mar 2 2020;98:109825. doi:10.1016/j.pnpbp.2019.109825
29. Liu Y, Chen J, Huang L, Yan S, Bian Q, Yang F. Relationships Among Retinal Nerve Fiber Layer Thickness, Vascular Endothelial Growth Factor, and Cognitive Impairment in Patients with Schizophrenia. Article. *Neuropsychiatric Disease and Treatment*. 2021;17:3597-3606. doi:10.2147/NDT.S336077
30. Orum MH, Bulut M, Karadag AS, Dumlupinar E, Kalenderoglu A. Comparison of OCT findings of schizophrenia patients using FGA, clozapine, and SGA other than clozapine. *Rev Psiquiatr Clin*. 2020;47(6):165-175. doi:http://dx.doi.org/10.15761/0101-60830000000257
31. Samani NN, Proudlock FA, Siram V, et al. Retinal Layer Abnormalities as Biomarkers of Schizophrenia. *Schizophr Bull*. Jun 6 2018;44(4):876-885. doi:10.1093/schbul/sbx130
32. Silverstein SM, Lai A, Green KM, Crosta C, Fradkin SI, Ramchandran RS. Retinal Microvasculature in Schizophrenia. *Eye Brain*. 2021;13:205-217. doi:10.2147/eb.S317186
33. Zhuo C, Xiao B, Chen C, et al. Abberant inverted U-shaped brain pattern and trait-related retinal impairment in schizophrenia patients with combined auditory and visual hallucinations: a pilot study. *Brain Imaging Behav*. Apr 2021;15(2):738-747. doi:10.1007/s11682-020-00281-y

34. Zhuo C, Xiao B, Ji F, et al. Patients with first-episode untreated schizophrenia who experience concomitant visual disturbances and auditory hallucinations exhibit co-impairment of the brain and retinas-a pilot study. *Brain Imaging Behav*. Jun 2021;15(3):1533-1541. doi:10.1007/s11682-020-00351-1
35. Zhuo C, Xiao B, Chen C, et al. Antipsychotic agents deteriorate brain and retinal function in schizophrenia patients with combined auditory and visual hallucinations: A pilot study and secondary follow-up study. *Brain Behav*. Jun 2020;10(6):e01611. doi:10.1002/brb3.1611
36. Zhuo C, Ji F, Xiao B, et al. Antipsychotic agent-induced deterioration of the visual system in first-episode untreated patients with schizophrenia maybe self-limited: Findings from a secondary small sample follow-up study based on a pilot follow-up study. *Psychiatry Res*. April 2020;286 (no pagination)112906. doi:http://dx.doi.org/10.1016/j.psychres.2020.112906
37. Silverman AL, Hammel N, Khachatryan N, et al. Diagnostic Accuracy of the Spectralis and Cirrus Reference Databases in Differentiating between Healthy and Early Glaucoma Eyes. *Ophthalmology*. Feb 2016;123(2):408-414. doi:10.1016/j.ophtha.2015.09.047
